# Exosomal Wnt7a from a low metastatic subclone promotes lung metastasis of a highly metastatic subclone in the murine 4t1 breast cancer

**DOI:** 10.1186/s13058-022-01557-5

**Published:** 2022-09-12

**Authors:** Chunning Li, Teizo Yoshimura, Miao Tian, Yuze Wang, Takamasa Kondo, Ken-Ichi Yamamoto, Masayoshi Fujisawa, Toshiaki Ohara, Masakiyo Sakaguchi, Akihiro Matsukawa

**Affiliations:** 1 Department of Pathology and Experimental Medicine, Graduate School of Medicine, Dentistry and Pharmaceutical Sciences, Okayama University, 2-5-1 Shikata, Kita-ku, Okayama, 700-8558 Japan; 2 Department of Cell Biology, Graduate School of Medicine, Dentistry and Pharmaceutical Sciences, Okayama University, 2-5-1 Shikata, Kita-ku, Okayama, 700-8558 Japan

**Keywords:** Breast cancer, Metastasis, Exosomes, Epithelial mesenchymal transition, Tumor microenvironment

## Abstract

**Background:**

Patients with triple-negative breast cancer (TNBC) often have poorer prognosis than those with other subtypes because of its aggressive behaviors. Cancer cells are heterogeneous, and only a few highly metastatic subclones metastasize. Although the majority of subclones may not metastasize, they could contribute by releasing factors that increase the capacity of highly metastatic cells and/or provide a favorable tumor microenvironment (TME). Here, we analyzed the interclonal communication in TNBC which leads to efficient cancer progression, particularly lung metastasis, using the polyclonal murine 4T1 BC model.

**Methods:**

We isolated two 4T1 subclones, LM.4T1 and HM.4T1 cells with a low and a high metastatic potential, respectively, and examined the effects of LM.4T1 cells on the behaviors of HM.4T1 cells using the cell scratch assay, sphere-forming assay, sphere invasion assay, RT-qPCR, and western blotting in vitro. We also examined the contribution of LM.4T1 cells to the lung metastasis of HM.4T1 cells and TME in vivo. To identify a critical factor which may be responsible for the effects by LM.4T1 cells, we analyzed the data obtained from the GEO database.

**Results:**

Co-injection of LM.4T1 cells significantly augmented lung metastases by HM.4T1 cells. LM.4T1-derived exosomes promoted the migration and invasion of HM.4T1 cells in vitro, and blocking the secretion of exosome abrogated their effects on HM.4T1 cells. Analyses of data obtained from the GEO database suggested that Wnt7a might be a critical factor responsible for the enhancing effects. In fact, a higher level of Wnt7a was detected in LM.4T1 cells, especially in exosomes, than in HM.4T1 cells, and deletion of Wnt7a in LM.4T1 cells significantly decreased the lung metastasis of HM.4T1 cells. Further, treatment with Wnt7a increased the spheroid formation by HM.4T1 cells via activation of the PI3K/Akt/mTOR signaling pathway. Finally, infiltration of αSMA-positive fibroblasts and angiogenesis was more prominent in tumors of LM.4T1 cells and deletion of Wnt7a in LM.4T1 cells markedly reduced angiogenesis.

**Conclusions:**

We demonstrated, for the first time, that a low metastatic subclone can enhance lung metastasis of highly metastatic subclone via exosomal Wnt7a and propose Wnt7a as a molecular target to treat TNBC patients.

**Supplementary Information:**

The online version contains supplementary material available at 10.1186/s13058-022-01557-5.

## Introduction

Tumor metastasis to distant organs is the main cause of mortality in breast cancer (BC) patients [1]. It is regulated by complicated processes that include the separation of tumor cells from the primary tumor, invasion into nearby blood and lymphatic vessels, extravasation from the vessel lumina into the parenchyma of distant organs, and subsequent growth from micrometastatic lesions into macroscopic tumors [2]. Triple-negative breast cancer (TNBC) is a subtype of breast cancer characterized by the absence of estrogen receptor, progesterone receptor, and HER2/ERBB2 expression and known for being aggressive and having a poor prognosis [3]. Because the therapeutic targets of TNBC are unknown, chemotherapy remains the mainstay of the treatment, even if some patients may not fully respond to chemotherapies [4]. Therefore, identifying a possible target for TNBC therapy is an urgent need.

Tumor cells are heterogeneous, and not all subclones have the capacity to metastasize. This notion is supported by the successful cloning of a few highly metastatic cells from human and mouse tumors [5]. Although the majority of subclones may not metastasize, they could contribute to the process by releasing tumor promoting factors that increase the capacity of highly metastatic cells and/or provide a favorable tumor microenvironment (TME). Several studies have shown that exosomes derived from TNBC cells promote the metastatic processes by transferring various information (i.e., RNA, DNA, and proteins) to recipient cells in original tumors or distant colonized organs [6, 7]. Exosomes are a subtype of extracellular vesicles produced during the maturation of reticulocytes [8], with a size of 30–150 nm and genesis in multivesicular bodies [9].

The Wnt/β-catenin signaling pathway has been demonstrated to be involved in cancer cell proliferation [10], metastasis [11], stemness maintenance [12], and cancer-associated systemic inflammation [13]. *Wnt1*, originally called *Int1*, is the first member of the Wnt family and was discovered as a potential cellular oncogene involved in mouse mammary tumor virus-induced mammary tumors [14]. There have been 19 distinct Wnt ligands identified in the last four decades. Wnt proteins bind receptors, such as Frizzled and/or low-density-lipoprotein receptor-related protein 5/6, and activate either the β-catenin-dependent (canonical) or independent (non-canonical) signaling pathways, and the expression of Wnt receptors is elevated in BC, especially in TNBC [15]. Exosomes were previously shown to transport Wnt ligands on their surface via the Evi exosomal protein and trigger signaling in target cells [16, 17]. Even though an increasing number of studies are focused on the impact of aberrant Wnt signaling in BC, no Wnt inhibitors have been utilized in treatment to date.

The murine 4T1 cell line is a murine TNBC cell line derived from a mammary tumor that spontaneously developed in a BALB/c mouse foster-nursed by a C3H female mouse (BALB/cfC3H) [18, 19]. In female BALB/c mice, 4T1 cells implanted at the orthotopic mammary fat pad spontaneously metastasize to multiple organs, including the bone, lung, and liver. 4T1 cells are also a mixture of subclones with different gene expression profiles and metastatic potentials [20]. Several studies have been performed to evaluate the heterogeneity and to classify various subpopulations among 4T1 cells based on cell properties, such as cellular morphology [21], cell proliferation, cancer stem cell-like [22], and metastatic ability [20]. Thus, 4T1 cells are an excellent model to explore the mechanisms of BC metastasis and study tumor cell heterogeneity. We previously observed that the levels of lung metastases by single-cell cloned 4T1 cells were significantly lower than those by parental 4T1 cells [23], leading us to the hypothesis that interactions among 4T1 subclones, potentially via secretion of exosomes, are essential for the maximal lung metastasis by highly metastatic 4T1 subclones.

In this study, we tested the hypothesis by establishing two 4T1 subclones with low metastatic (LM) and high metastatic (HM) capacity and performing a series of experiments both in vitro and in vivo. Our study demonstrates, for the first time, that LM.4T1 cells have the capacity to enhance the metastatic ability of HM.4T1 cells via exosomal Wnt7a and Wnt7a is potentially a target for the treatment of TNBC patients. Our results also indicate that interclonal communications among cancer cell subclones play an import role in cancer progression.

## Materials and methods

### Cell lines

Murine breast cancer 4T1 cell line was obtained from American Type Culture Collection (ATCC, USA). The parental 4T1 and single-cell clone cells were grown in RPMI 1640 medium (Sigma-Aldrich, USA), supplemented 10% fetal bovine serum (FBS) (HyClone, USA), 100 μM glutamine, 100 U/ml penicillin, 100 μg/ml streptomycin, and 1 mM sodium pyruvate (Wako, Japan) (complete medium).

### Antibodies

Antibodies used in this study are listed in Additional file 1: Table S1.

### Conventional RT-PCR and Real-time quantitative RT-PCR

For gene expression analysis, total RNA was extracted from cells and tumor tissues by using High Pure RNA Isolation Kit (Roche, Mannheim, Germany) or TRI^TM^sure (Nippon Genetics, Tokyo, Japan) reagent. The complementary DNA (cDNA) was synthesized with the High-Capacity cDNA Reverse Transcription Kit (ThermoFisher, Waltham, MA). KOD FX DNA polymerase (Toyobo, Osaka, Japan) was used in conventional RT-PCR for detection of Wnt7a expression in LM.4T1 and HM.4T1 cells. Annealing temperature was 57℃ for Wnt7a and β-actin. The primers sequences were as follows: Wnt7a: forward, 5′-GACAAATACAACGAGGCCGT-3′, reverse, 5′-GGCTGTCTTATTGCAGGCTC-3′. β-actin: forward, 5′-CAGCTGAGAGGGAAATCGTG-3′, reverse, 5′-CGTTGCCAATAGTGATGACC-3′. Real-time quantitative RT-PCR was performed using the Applied Biosystems *Step* *One*™ *Real*-*Time* *PCR* System (Life Technologies, Gaithersburg, MD). The expression of the *Il6*, *Il1b*, *Tgfb1*, *Tnfa*, *Vegfa*, *Hgf*, and *Cola1a* genes was analyzed by Taqman gene expression assay (Applied Biosystems, Foster City, CA). The total volume of each reaction was 10 µl containing 5.2 µl TaqManTM ProAmpTM Master Mix reagent, 0.4 µl primer and 2.8 µl UltraPure™ DNase/RNase-Free Distilled Water (Invitrogen, Carlsbad, CA). The primers of *Wnt7a* with probe for real-time PCR assay were purchased from Integrated DNA Technologies (Singapore, Republic of Singapore). The expression level of each gene was normalized to that of the *Gapdh* gene and presented as fold change over the expression of the control gene.

### Western blotting

For total protein isolation, cells were lysed in a lysis buffer (Cell Signaling, Danvers, MA) containing protease inhibitor cocktail (Roche) and Halt™ Phosphatase inhibitor cocktail (ThermoFisher), incubated on ice for 30 min, and then centrifuged at 14,500×*g* for 10 min. Exosomes were resuspended in radioimmunoprecipitation assay (RIPA) buffer for western blot analysis. Tumor tissues were smashed in RIPA buffer containing phenylmethanesulfonyl fluoride. For assessment of β-catenin activation, nuclear and cytoplasmic extracts were prepared using a NE-PER Nuclear and Cytoplasmic Extraction Kit (ThermoFisher) according to the manufacturer’s instructions. The protein concentrations were measured by BCA Protein Assay Kit (Takara, Tokyo, Japan). Equal amounts of protein samples were denatured at 100℃ for 10 min with 4 × NuPAGE LDS sample buffer and 10 × Sample Reducing agent (Invitrogen). Proteins (3 μg for exosome, 15–30 μg for cell lysates) were separated by 4–12% NuPAGE Bis–Tris precast gel (ThermoFisher) and transferred onto nitrocellulose blotting membranes (GE Healthcare Life science, Freiberg, Germany). The membranes were blocked with 5% milk in tris-buffer saline-Tween 20 for 1 h with at room temperature. After overnight incubation with a primary antibody at 4℃, the membrane was washed with TBS-T for 10 min three times, and then incubated with horseradish peroxidase (HRP)-conjugated secondary antibodies for 1 h at room temperature. After secondary antibody incubation, the membranes were washed again for 10 min three times and the presence of the protein of interest was visualized and quantitated with C-DiGit Blot scanner (Scrum, Tokyo, Japan).

### Sphere formation and spheroid invasion assay

For spheroid generation, 1 or 2 × 10^3^ cells were seeded in ultra-low attachment 96-well round-bottomed plates (Corning, Corning, NY) with sphere-forming medium DMEM/F12 (Invitrogen) supplemented with 1 × B27 serum substitute (Invitrogen), 20 ng/ml mouse recombinant epidermal growth factor (PeproTech, Rocky Hill, NJ) and 10 ng/ml basic fibroblast growth factor (PeproTech). HM.4T1 spheroid were stimulated with 100 ng/ml recombinant human Wnt7a protein (Abcam, UK) or 100 ng/ml recombinant mouse Wnt3a protein (BioLegend, San Diego, CA). To inhibit PI3K or mTOR, spheroids were pre-treated for 1 h with 10 μM LY294002 or 50 nM rapamycin, and then, 100 ng/ml Wnt7a was added. The size of spheroids was evaluated at 24 h, 48 h, and 72 h. Cell lysates were prepared at 6 h or 24 h after addition of Wnt7a. The radius of each spheroid was measured by using the Image J software, and the volume (μm^3^) was calculated by the following formula: V = 4/3*πr*^3^.

We performed an invasion assay using the generated tumor spheroid. After 4 days of culture, the sphere-forming medium in each well was aspirated and switched to 100 μl Matrigel Matrix (Corning). Each spheroid was ensured to be in the center of the well. After 1-h incubation at 37 °C, each well was covered with 100 μl sphere-forming medium. The invasion area was monitored by IX2-SLP OLYMPUS microscope (Olympus, Tokyo, Japan), captured and measured by using the Image J software.

### Immunofluorescence

One thousand cells were seeded into a Lab-Tek II chamber slide (Nalge Nunc, Rochester, NY). When cells became 60% confluent, they were stimulated with 100 ng/ml recombinant human Wnt7a protein or 100 ng/ml recombinant mouse Wnt3a protein. After 24-h incubation, medium was aspirated, and the cells were fixed in 4% paraformaldehyde for 15 min at room temperature. Cells were washed with phosphate-buffered saline (PBS) 5 min for three times, permeabilized with 0.5% Triton X-100 at room temperature for 20 min, washed with PBS 5 min three times, blocked with PBS containing 1% bovine serum albumin for 1 h at room temperature, and incubated with anti-β-catenin Ab at 4℃ overnight. After washing with PBS containing 0.1% Tween^TM^20 for 5 min three times, cells were incubated with Alexa Fluor 488-conjugated secondary antibody in the darkness for 1 h at room temperature. After washing for 5 min three times, slides were mounted using the ProLong™ Gold antifade mountant with DAPI (ThermoFisher). Images were acquired using fluorescence microscope BZ-X700 (Keyence, Tokyo, Japan).

### Immunohistochemistry

Tumors were fixed overnight in 10% formalin and embedded in paraffin. Immunostaining was performed manually by a conventional method: Briefly, sections were deparaffinized in xylene and rehydrated in a sequence of descending concentrations of ethanol. Endogenous peroxidase reactivity was blocked with 3% H_2_O_2_ for 10 min. For antigen retrieval, sections were submerged in 10 mM citrate buffer (pH6.0) or 5 mM EDTA solution (pH8.0) and microwaved (700 W) continuously for 15 min in a pressure cooker and then incubated with a respective primary antibody for 1.5 h at room temperature. After washing, sections were incubated with an appropriate secondary antibody conjugated with horseradish peroxidase (Nichirei, Tokyo, Japan) and the signals were visualized using DAB (Dako, Santa Clara, CA) according to the manufacturer’s instructions. Finally, sections were counterstained with hematoxylin, dehydrated, and mounted. Images were acquired using an Olympus BX43 light microscope connected to a DP73 digital camera (Olympus).

### Exosome isolation

Cells were grown to 80% confluent in complete medium and then in RPMI 1640 medium containing 2% exosome-depleted FBS for 48 h. The medium containing exosomes was collected and centrifuged for 10 min at 500×*g*, 20 min at 2000×*g*, and 30 min at 10,000×*g*. The exosomes pellet was harvested after 70 min of ultracentrifugation at 100,000×*g* (Beckman, Fullerton, CA) and then washed with PBS and ultracentrifugation again at 100,000×*g* for 70 min. Exosomes were then resuspended in PBS or RIPA for further studies. The concentration of exosomes suspension was measured by using Pierce™ BCA protein assay kit (Takara).

### Transmission electron microscopy (TEM)

Exosomes were resuspended in PBS to be used for TEM analysis. Hydrophilic treatment was performed on a 400-mesh copper grid coated with formvar/carbon films. Exosome suspension (10 μl) was placed on Parafilm, and the grid was floating on top of the exosome suspension was left for 15 min. The sample was negatively stained with 2% uranyl acetate solution for 2 min. The grids were washed five times with PBS after each staining step and finally ten times in H_2_O before contrast staining with 2% uranyl acetate solution. Exosomes on the grid were imaged at 20,000 times magnifications using an H-7650 transmission electron microscope (Hitachi, Tokyo, Japan) in the Central Research Laboratory, Okayama University Medical School.

### Cell scratch assay

Cell scratch assay was used to evaluate cell migratory ability. Cells were seeded in a 6-well plate and cultured to 80% confluent. Cell monolayer was scratched with a sterile 1000 μl pipette tip and washed twice with PBS, and floated cells were aspirated. Finally, 2 ml of fresh medium was added to each well and cell monolayer was photographed with a microscope. Cell migratory ability was monitored for HM.4T1 cells at 24 h, 48 h, and 72 h. Using Image J software, wound recovery (%) = 100(*A*–*B*)/*B* was calculated, with A and B indicating the area of cell scratches before and after incubation, respectively.

### Cell proliferation assay

In a 96-well microplate, cells were seeded at a density of 2 × 10^3^ cells per well. After 4 days of culture, 10 μl of MTT labeling reagent (Roche) was added to each well. After a 4-h incubation in a humidified environment, 100 μl of the solubilization solution (Roche) was added to each well. Plate was allowed to stand overnight in an incubator in a humidified atmosphere (+ 37 ℃, 5–6.5% CO_2_). After complete solubilization of the purple formazan crystals, the spectrophotometrical absorbance of the samples was measured using a microplate reader. The wavelength to measure the absorbance of the formazan product was between 570 and 690 nm.

### Generation of green fluorescent protein (GFP)- or red fluorescent protein (RFP)-expressing cells

LM.4T1 or HM.4T1 cells were transfected with GFP or RFP expression vector [24] using Neon™ Transfection system (ThermoFisher) and then incubated in complete medium containing 20 μg/ml puromycin for GFP-expressing cells or 5 μg/ml blasticidin for RFP-expressing cells (InvivoGen, San Diego, CA).

### Generation of Wnt7a- and Rab27a-deficient cell lines by CRISPR/Cas9 system

To stably delete the expression of Wnt7a or Rab27a in LM.4T1 and HM.4T1 cells, cells were transfected with each targeting vector using Neon™ Transfection system. We designed three sgRNA oligonucleotides for each gene and tested their effects on gene silencing. All sgRNA oligonucleotides were against the two genes, and one of each oligonucleotide was chosen for the following experiments. The sgRNA sequence for Wnt7 gene was 5′-TCCGGAGGTAGACTATGCCC-3′. The sgRNA sequence for Rab27a gene was 5′-CCACCTGCAGTTATGGGACA-3′. Seven days after transfection, medium was changed to antibiotic-containing selection medium. When single-cell clone cells grew, several clones were picked, and protein was extracted for western blot analysis. The clones that lost the expression of targeted gene were also verified by DNA sequencing. Wnt7a-deficient and Rab27a-deficient cells were grown in complete medium containing 20 μg/ml puromycin.

### Tumor transplantation model

Female BALB/c mice were purchased from Japan SLC, Inc. (Hamamatsu, Japan). MD. One million tumor cells were seeded in a T-75 tissue culture flask and grown to 50–80% confluence. Cells were detached with 0.2% trypsin–EDTA, washed once with complete medium and three times with PBS, and resuspended in PBS at 1 × 10^6^ cells/ml. One hundred and thousand cells in 100 μl PBS were injected into the third left mammary pad. Tumor tissues were excised and fixed in 10% formalin. Lungs were perfused with Bouin’s solution (Wako, Osaka, Japan) and fixed in the same solution, and then, the number of tumor nodules was counted by eye. After fixation in Bouin’s solution, subpleural lung surface metastases were easily identified by their light, white appearance. Tumor length and width were measured using a caliper, and tumor volume was calculated using the following formula: Volume = (width)^2^ × length/2.

LM.4T1-RFP and HM.4T1-GFP cells were implanted into nude (BALB/c-nu/nu) mice (Japan SLC, Inc.). Four weeks later, lungs were removed and metastases foci of GFP and RFP expression cells were immediately visualized using a fluorescence microscope (Olympus stereoscopic microscope, SZX12). Lungs were then fixed in Bouin's solution to detect tumor nodules. LM-Wnt7a KO, HM-Wnt7a KO, and LM-Rab27a KO cells were also implanted into the mammary pad of BALB/c-nu/nu mice.

### Analysis of database

The ID of the RNA sequencing data of 23 4T1 subclones from the Gene Expression Omnibus (GEO) database is GSE63180 [20], which we then re-analyzed using a web-application GREIN (GEO RNA-seq Experiments Interactive Navigator), and GREIN is accessible at: https://shiny.ilincs.org/grein [25]. We utilized the Kaplan–Meier Plotter database for survival analysis, which is available at https://kmplot.com/analysis/ [26].

### Statistical analysis

Results were analyzed by the GraphPad Prism 9.0 software (San Diego, CA) and presented as the mean ± standard error of mean (SEM). Each experiment was carried out in technical and biological triplicate. The Student’s t test was used to compare two groups. One-way ANOVA and two-way ANOVA were used to determine multiple group comparisons. A value of *p* < 0.05 was deemed statistically significant.

## Results

### Establishment of two 4T1 subclones, LM.4T1 and HM.4T1 with a low and high metastatic potential

To study whether lung metastasis of highly metastatic subclones could be further enhanced by low metastatic subclones, we first isolated several 4T1 subclones from parental 4T1 cells by limiting dilution (Additional file 1: Fig. S1). Morphologically, there were two types of clones when cultured in 6-well tissue culture plates: One showed rod shape and grew as cell islands (Type A), and the other was more adherent, spread well, and grew as cell sheets (Type B). We selected 3 clones from each group: clones 4T1-A8, C1, and E2 from the Type A group and clones C8, F3, and F9 from the Type B group (Additional file 1: Fig. S1A). We implanted these 6 clones into the mammary pad of BALB/c mice to assess their local growth and metastatic potential. Tumors of 4T1-F3 and F9 appeared to grow more quickly than other clones, but there was no statistical significance (Additional file 1: Fig. S1B and S1C). The metastatic potential of all 4T1 subclones was significantly lower than that of parental cells. Among the subclones, 4T1-C1 in the Type 1 group showed the highest metastatic potential (Additional file 1: Fig. S1D), whereas 4T1-F3 in the Type 2 group showed the lowest. We performed another round of limiting dilution and subsequently obtained two single-cell clones, termed low metastatic (LM) 0.4T1 from 4T1-F3 and high metastatic (HM) 0.4T1 from 4T1-C1 for further characterization.

**Fig. 1:**
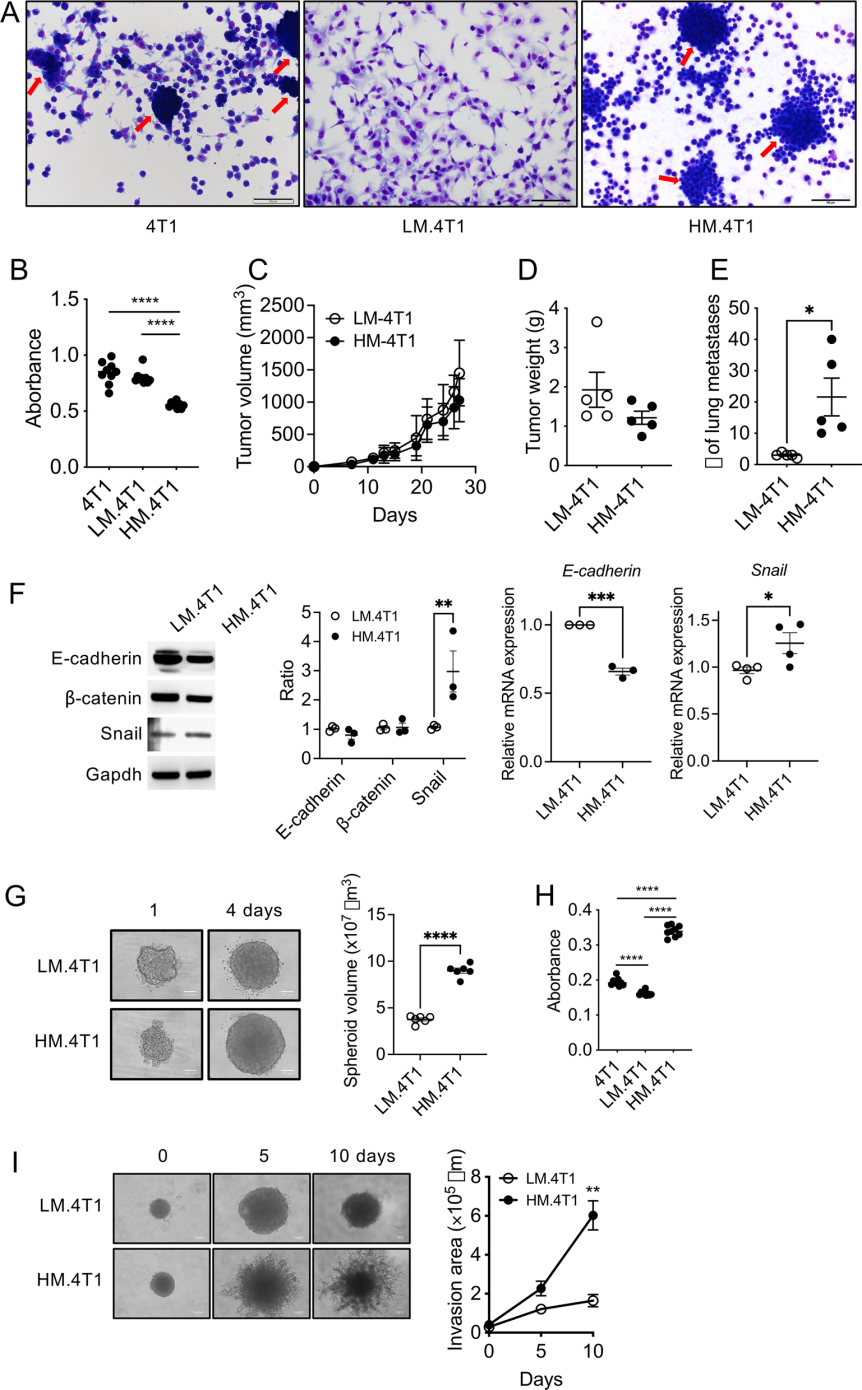
Single-cell clones derived from parental 4T1 cells exhibit varying characteristics. **A** One thousand 4T1, LM.4T1, or HM.4T1 cells were seeded into a 8-well Lab-Tek II chamber slide and cultured for 4 days at 37 °C and then stained with Diff-Quik. The photographs show distinct morphologies of LM.4T1 and HM.4T1 cells. Arrows indicate spheroids. The scale bar indicates 100 μm. **B** One thousand 4T1, LM.4T1, or HM.4T1 cells were seeded into 96-well plates and allowed to grow for four days. The cell numbers were determined by the standard MTT assay. The results are presented as the mean ± SEM. Statistical significance was analyzed by one-way ANOVA with multiple comparisons. ****p < 0.0001, *n* = 9. **C** LM.4T1 and HM.4T1 cells (1 × 10^5^ cells in 100 μl PBS) were transplanted into the 3^rd^ mammary pad of BALB/c mice, and the sizes of tumors were measured, and tumor volumes were calculated. The results are presented as the mean ± SEM. *n* = 5. **D**, **E** Mice were euthanized 4 weeks after injection, and the weight of tumors and the number of lung metastases were evaluated. The results are presented as the mean ± SEM. Statistical significance was analyzed by unpaired Student’s *t* test. *p < 0.05, *n* = 5. **F** LM.4T1 and HM.4T1 cells were cultured in 6-well plates. When cells became 80% confluent, total protein and total RNA were isolated. The levels of E-cadherin, $\upbeta$-catenin, and Snail protein were examined by western blotting (*n* = 3). The levels of *E-cadherin* (*n* = 3) and *Snail* mRNA (*n* = 4) were examined by RT-qPCR. The results are presented as the mean ± SEM. Statistical significance was analyzed by two-way ANOVA with multiple comparisons. **p < 0.05. **G** Sphere formation assay using LM.4T1 and HM.4T1 cells. For sphere generation, one thousand cells were seeded into 96-well ultra-low attachment plate and grow for 4 days. The diameter of each sphere measured by using the Image J software and the volume of each sphere were calculated. The scale bar indicates 50 μm. The results are presented as the mean ± SEM. Statistical significance was analyzed by unpaired Student’s *t* test. ****p < 0.0001, *n* = 6. **H** One thousand 4T1, LM.4T1, or HM.4T1 cells were cultured in 96-well ultra-low attachment plates, and the growth of each cell type was compared by MTT assay. The results are presented as the mean ± SEM. Statistical significance was analyzed by one-way ANOVA with multiple comparisons. ****p < 0.0001, *n* = 9. **I** The ability of cell migration and invasion in 3D was examined by sphere invasion assay. After four-day culture to form spheroids (day 0), medium was replaced with Matrigel and the invasion of cells was observed on day 5 and 10. The invasion area was measured by using the Image J software. The results are presented as the mean ± SEM. Statistical significance was analyzed by unpaired Student’s *t* test. **p < 0.001, *n* = 5. The scale bar indicates 100 μm

When parental 4T1, LM.4T1, and HM.4T1 cells were cultured in LAB-TEK chambers, parental 4T1 cells were found to comprise at least two types of cells: One type of cells grew in cell sheets, whereas the other formed a sphere-like structure (red arrows) with a lower ability to adhere and spread. LM.4T1 appeared epithelial cell-like, whereas HM.4T1 appeared mesenchymal cell-like (Fig. 1A). In an in vitro cell proliferation assay, HM.4T1 cells proliferated slower than either parental 4T1 or LM.4T1 cells (Fig. 1B). In vivo, however, the volumes of both LM.4T1 and HM.4T1 tumors increased at a similar rate (Fig. 1C, D). As expected, larger numbers of lung metastases were found with HM.4T1 cells than with LM.4T1 cells (Fig. 1E). By western blotting, there was no significant difference in the expression of E-cadherin or β-catenin between the two subclones, but HM.4T1 cells expressed a higher level of Snail than LM.4T1 cells (Fig. 1F). By RT-qPCR, HM.4T1 cells expressed a lower level of *E-cadherin* mRNA and a higher level of *Snail* mRNA than LM.4T1 cells (Fig. 1F). Thus, HM.4T1 cells appeared to be in a more advanced EMT stage.

We examined the behaviors of the two subclones in 3D culture using sphere-forming and sphere invasion assays, widely used model that may better mimic tumor cell behaviors in vivo [27]. By sphere-forming assay, HM.4T1 cells formed significantly larger spheres than LM.4T1 cells after 1-day or 4-day culture (Fig. 1G) and the proliferation of HM.4T1 cells was significantly higher than that of LM.4T1 cells by MTT assay (Fig. 1H). By sphere invasion assay, HM.4T1 cells invaded more aggressively than LM.4T1 cells (Fig. 1I). Thus, parental 4T1 cells consist of at least two groups of subclones with a distinct morphological feature and a metastatic potential.

### Co-injection of LM.4T1 increases the lung metastasis of HM.4T1 cells

**Fig. 2:**
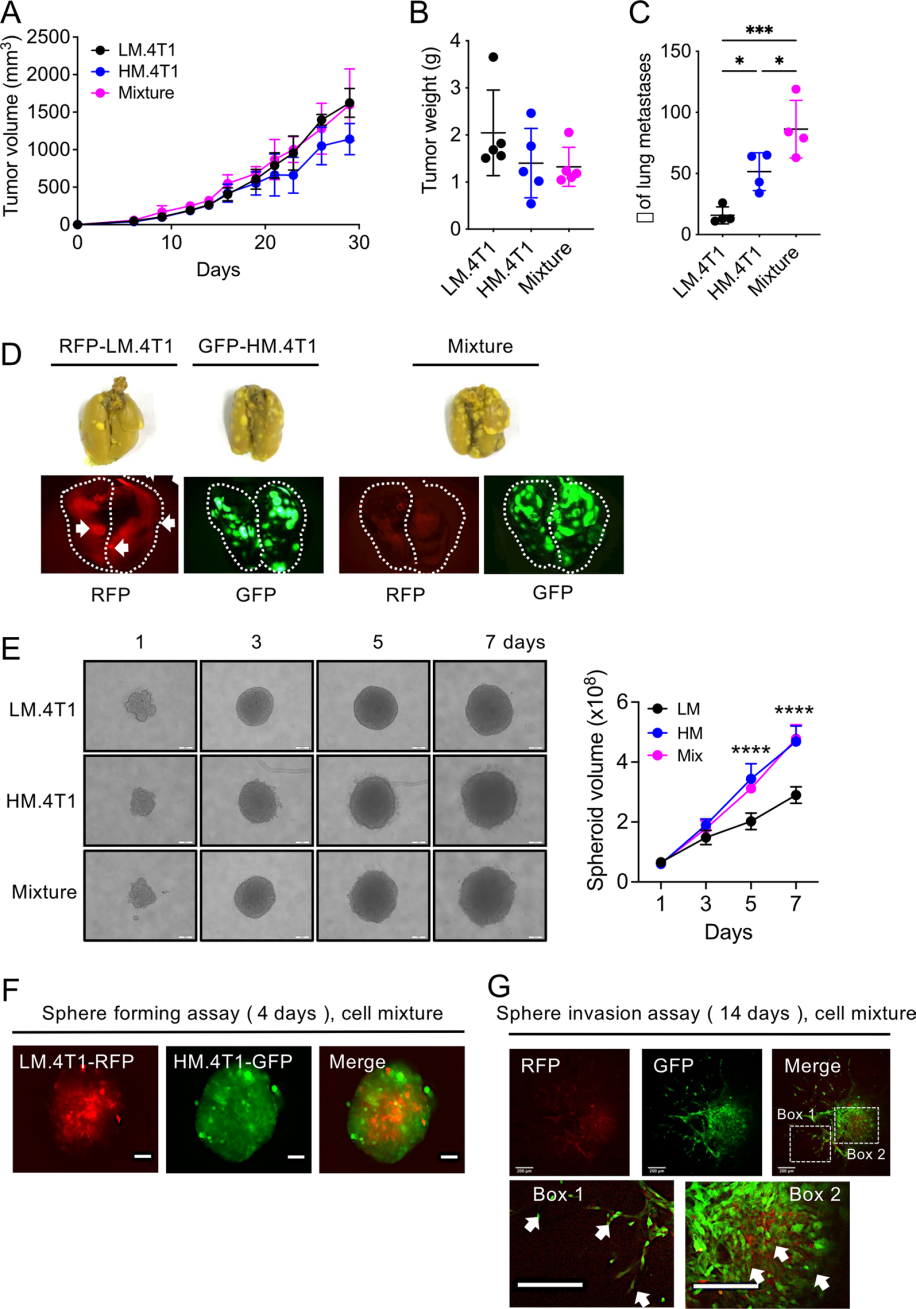
Co-injection of LM.4T1 augmented the lung metastasis of HM.4T1 cells. **A** One hundred and thousand LM.4T1, HM.4T1 cells, or 1:1 cell mixture were injected into the 3rd mammary pad of BALB/c mice. Tumor sizes were monitored for 28 days, and the volume of tumors was calculated. The results are presented as the mean ± SEM. *n* = 5. **B** After 4 weeks of tumor growth, mice were euthanized, and tumor weights were measured. The results are presented as the mean ± SEM. *n* = 5. **C** The number of metastatic tumor nodules in the lung was quantified and compared among three different groups. The results are presented as the mean ± SEM. Statistical significance was analyzed by one-way ANOVA with multiple comparisons. *p < 0.05, ***p < 0.001, *n* = 4. **D** One hundred and thousand 1:1 cell mixture of RFP-labeled LM.4T1 cells and GFP-labeled HM.4T1 cells was injected into the 3rd mammary pad of BALB/c nude mice. Four weeks after the injection, metastasized cells in lung tissues were examined under a fluorescent microscope (Olympus stereoscopic microscope SZX12). Arrows indicate three tumors detected on lung surface. Photographs of Bouin’s solution-fixed lungs are also presented. **E** Two thousand cells were allowed to grow for 7 days in 96-well ultra-low attachment plates. The diameter of each spheroid was measured by Image J software, and the volume of each spheroid was calculated. The scale bar indicates 100 μm. The results are presented as the mean ± SEM. Statistical significance was analyzed by unpaired Student’s *t* test. ****p < 0.0001. *n* = 5. **F** One thousand 1:1 cell mixture of LM.4T1-RFP cells and HM.4T1-GFP cells was allowed to grow for 4 days in 96-well ultra-low attachment plates. The localization of each cell type was examined under a fluorescence microscope (KEYENCE, All-in-one fluorescence microscope BZ-X700). Scale bar indicates 100 μm. **G** Medium was replaced with Matrigel, and the cells were cultured for an additional 10 days. Cells in the invading front (box 1) and in the center of spheroids (box 2) were examined using a confocal microscope (Carl Zeiss Confocal Laser Scanning Microscope LSM780). Scale bar indicates 200 μm

To examine interclonal cooperation between LM.4T1 and HM.4T1 cells in BC progression, we injected 1 × 10^5^ LM.4T1, HM.4T1, or 1:1 cell mixture into BALB/c mice. As shown in Fig. 2A, B, there were no significant differences in local tumor growth among three groups; however, the number of lung metastases was significantly higher when the cell mixture was injected (Fig. 2C). Since this experiment did not allow us to determine which subclone cells metastasized to the lung, we tagged LM.4T1 and HM.4T1 cells with RFP and GFP, respectively, and then implanted each tagged subclones or cell mixture (1:1) into nude (BALB/c-nu/nu) mice. We chose nude mice because the tagged cells failed to grow in BALB/c mice likely due to the immune response. Four weeks later, we excised the lungs and examined the presence of tagged cells under a fluorescent microscope. A higher number of lung metastases were detectable in mice injected with GFP-HM.4T1 than with RFP-LM.4T1 cells. In mice that received the cell mixture, most of metastasized cells in the lung were GFP-positive, and RFP-positive cells were hardly detected in the same lung (Fig. 2D). This result strongly suggested that increased lung metastases found by the co-injection of LM.4T1 and HM.4T1 cells were due to an increased level of metastases by HM.4T1 cells.

We studied the cooperation between the two subclones in more detail using sphere-forming and sphere invasion assay in vitro. By sphere-forming assay, the size of HM.4T1 spheres was significantly larger than that of LM.4T1 spheres, and the size of spheres formed by the cell mixture was same as that of HM.4T1 spheres (Fig. 2E). To examine the distribution of each subclone cells in the spheres, we co-cultured GFP-HM.4T1 with RFP-LM.4T1 cells and found that RFP-LM.4T1 cells were mostly inside the spheres and GFP-HM.4T1 cells were outside the spheres (Fig. 2F). By sphere invasion assay, GFP-HM.4T1 cells were found in the invading front (Fig. 2G). These data suggested that HM.4T1 cells have a stronger anchorage-independent cell growth and invasion capacity than LM.4T1 cells.

### Exosomes derived from LM.4T1 cells augment the migration and invasion of HM.4T1 cells

**Fig. 3:**
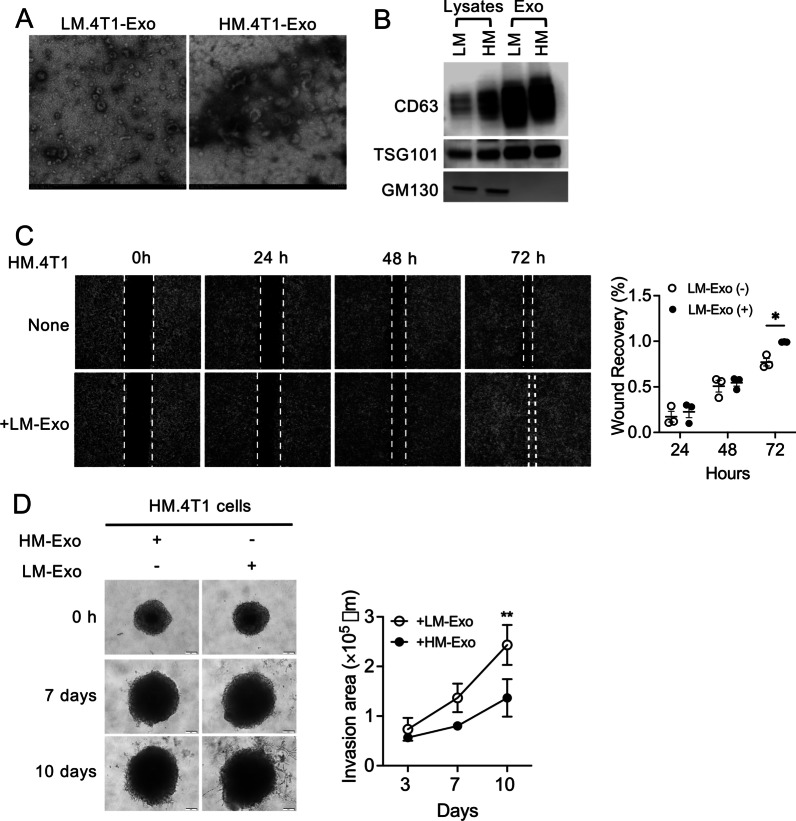
Exosomes derived from LM.4T1 cells can promote HM.4T1 cell migration and invasion. **A** Transmission electron microscopic images of exosomes purified from the culture supernatant of LM.4T1 or HM.4T1 cells. The scale bar indicates 200 nm. **B** The presence of exosome-enriched protein markers, including CD63 and TSG101, as well as the absence of the negative exosome marker GM130 in cell lysates and exosomes of LM.4T1 and HM.4T1 cells was analyzed by western blotting. **C** Wound healing assay was used to investigate the migration of HM.4T1 cells co-cultured with or without exosomes from LM.4T1 cells. The results are presented as the mean ± SEM. Statistical significance was analyzed by two-way ANOVA with multiple comparisons. *p < 0.05, *n* = 3. **D** Spheroid invasion assay was used to evaluate the invasion of HM.4T1 cells co-cultured with LM.4T1-derived exosomes. The results are presented as the mean ± SEM. Statistical significance was analyzed by unpaired Student’s *t* test. **p < 0.01. *n* = 3. The scale bar indicates 100 μm

Exosomes derived from TNBC cells have been shown to promote cancer cell metastasis by transferring molecules (i.e., RNA, DNA, and proteins) to recipient cells in original tumors [6, 7]. To examine whether exosomes play a role in the interclonal communication, we isolated exosomes from the culture supernatants of LM.4T1 or HM.4T1 cells by ultracentrifugation. The typical cup shape of exosomes was seen by TEM (Fig. 3A). Western blotting confirmed that CD63 and TSG101, markers for extracellular vesicles, were enriched in exosome fractions, whereas the Golgi marker GM130 was detected only in cell lysates (Fig. 3B). We cultured HM.4T1 cells in the presence or absence of exosomes from LM.4T1 cells and evaluated its effects on the behavior of HM.4T1 cells in the cell scratch and sphere invasion assay in vitro. Addition of LM.4T1 cell-derived exosomes promoted HM.4T1 cell wound closing (Fig. 3C). It also increased the migration of HM.4T1 cells in a transwell assay (Additional file 1: Fig. S2A), but not the proliferation of HM.4T1 cells (Additional file 1: Fig. S2B). Addition of exosomes from LM.4T1 cells, but not HM.4T1 cells, also increased HM.4T1 cell invasion (Fig. 3D). These results suggested that exosomes derived from LM.4T1 cells augment the migration and invasion of HM.4T1 cells.

**Fig. 4:**
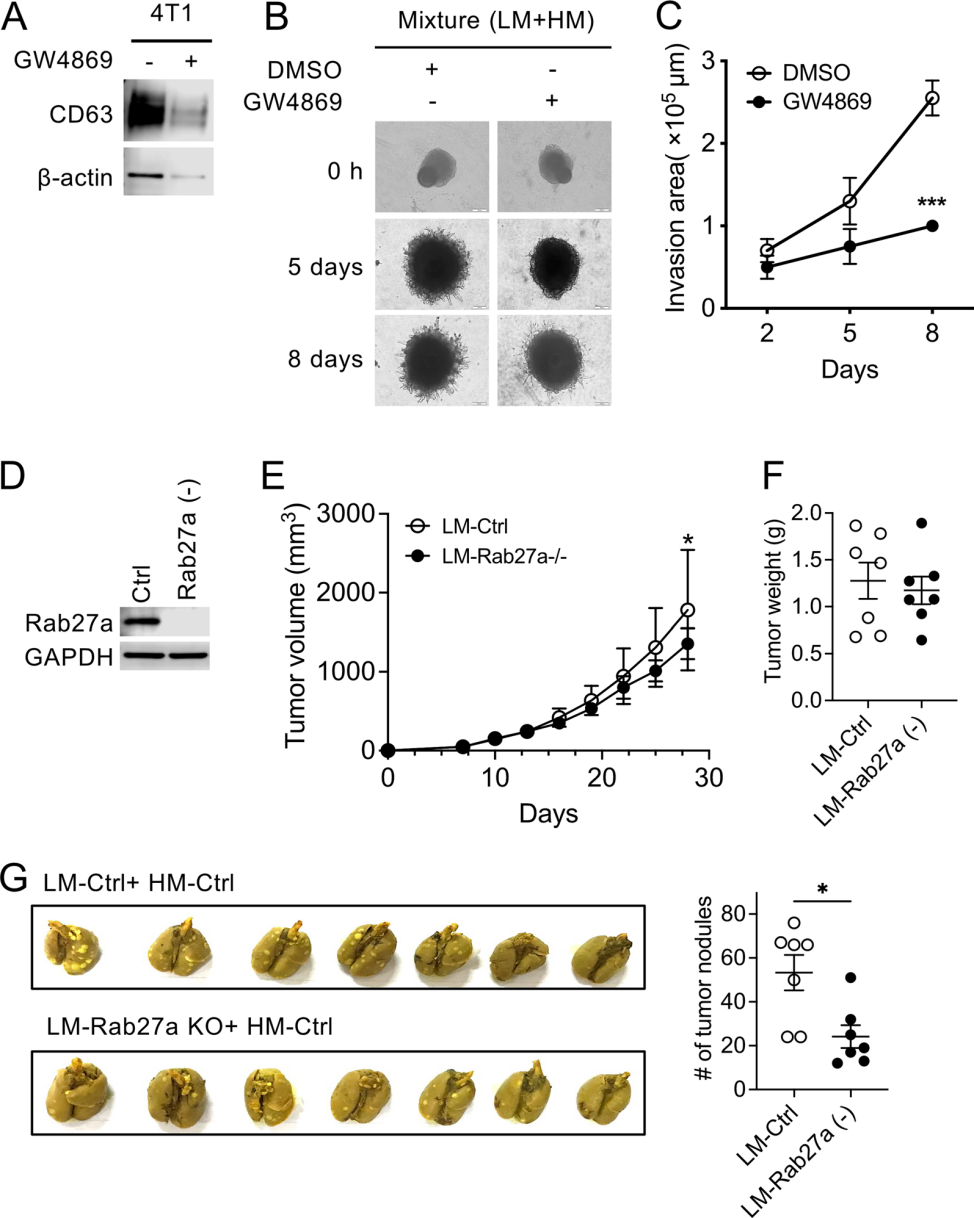
Inhibition of exosome production in LM.4T1 cells decreased the lung metastasis of HM.4T1 cells. **A** Parental 4T1 cells were treated for 48 h with the exosome biogenesis inhibitor GW4869. Exosomes were isolated from 50 ml culture supernatant of 4T1 cells either GW4869-treated or untreated. The presence of the exosome-enriched protein marker CD63 was examined in each sample using Western blotting. **B** LM.4T1 cells were mixed with HM.4T1 cells 1:1 and incubated for 4 days in regular medium. The medium was then replaced by 100 μl Matrigel containing DMSO or GW4869 and incubated for an additional 8 days. The photographs were taken on day 5 and day 8. The scale bar indicates 100 μm. **C** The invasion areas were measured by using Image J software. The results are presented as the mean ± SEM. Statistical significance was analyzed by unpaired Student’s *t* test. ***p < 0.001. **D** CRISPR-Cas9 was utilized to mutate the *Rab27a* gene in LM.4T1 cells. The loss of Rab27a protein was confirmed by western blotting. **E** Fifty thousand control LM.4T1 cells or Rab27a (−) LM.4T1 cells were mixed with the same number of HM.4T1 cells (1:1) and implanted into the 3rd mammary pad of BALB/c nude mice. The tumor size was measured, and the tumor volume was calculated. The results are presented as the mean ± SEM. Statistical significance was analyzed by unpaired Student’s t test. **p < 0.01, *n* = 7. **F** Mice were euthanized on day 28, and the weight of tumor was measured. The results are presented as the mean ± SEM. Statistical significance was analyzed by unpaired Student’s *t* test. *n* = 7. **G** The images of lung tissues with metastatic nodules are presented (left panel). The number of lung metastases was counted (right panel). The results are presented as the mean ± SEM. Statistical significance was analyzed by unpaired Student’s t test. *p < 0.05, *n* = 7

To obtain additional evidence indicating the involvement of exosomes, we used GW4869, a well-known inhibitor of neutral sphingomyelinase, to block exosome production [28]. Treatment with GW4869 significantly decreased the secretion of exosomes by parental 4T1 cells (Fig. 4A). When a mixture of LM.4T1 and HM.4T1 cells was co-cultured in the presence of GW4869, the invasion area was significantly decreased in sphere invasion assay (Fig. 4B, C). We also mutated the gene for Rab27a, a molecule regulating exosome production by 4T1 cells [29], in LM.4T1 cells by using the CRISPR/Cas9 technology. The presence of mutation in the *Rab27a* gene and the absence of Rab27a in LM.4T1-Rab27a-deficient cells were confirmed by DNA sequencing (Additional file 1: Fig. S3) and western blotting (Fig. 4D), respectively. We co-injected HM.4T1 cells with Rab27a (+) LM.4T1 cells or Rab27a (−) LM.4T1cells in nude mice and compared the local tumor growth and lung metastasis. The volume of tumors formed by the co-injection of Rab27a (−) cells was slightly lower than that by Rab27a (+) LM.4T1 cells on day 28 (Fig. 4E), but there was no difference in tumor weight (Fig. 4F). The number of metastases in the lung was significantly reduced when Rab27 (−) cells were co-injected (Fig. 4G). These findings further suggested that exosomes secreted from LM.4T1 cells promoted lung metastasis of HM.4T1 cells.

### Identification of Wnt7a as a potential LM.4T1 exosomal protein

**Fig. 5:**
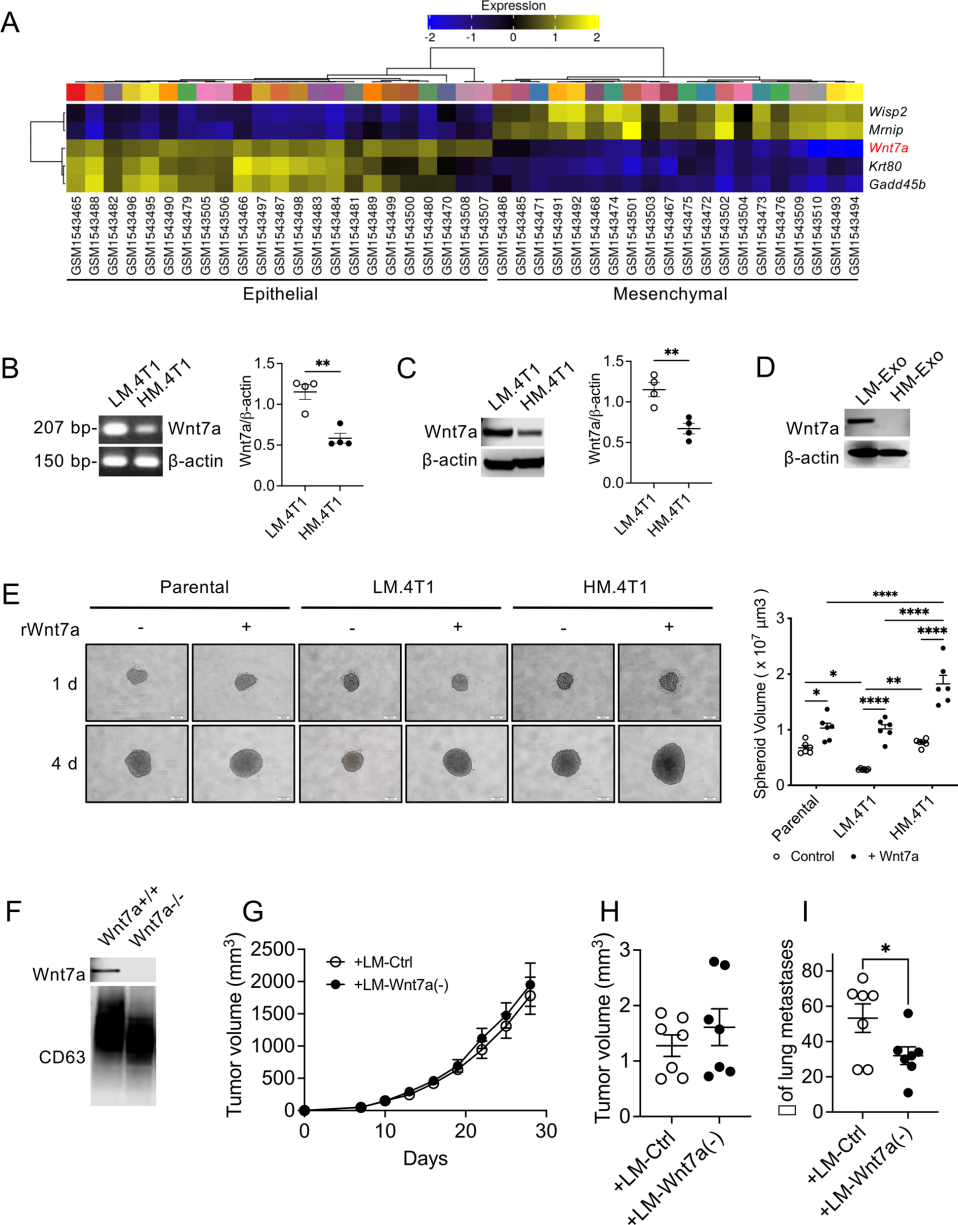
Wnt7a is a possible candidate protein that plays a role in the enhanced HM.4T1 cell lung metastasis by LM.4T1 cells. **A** A heat map was generated using normalized RNA-seq data (ID: GSE63180) (Wagenblast et al., 2015) that we obtained from the NCBI GEO (Gene Expression Omnibus, National Center for Biotechnology Information, US National Library of Medicine) database. Gene enrichment analysis in 23 clones from 4T1 cells separated in two subgroups: One displays epithelial features and another displays mesenchymal features. The image shows top five different expression genes between two different groups (ranked by adjusted *p* values). **B** *Wnt7a* mRNA expression in LM.4T1 and HM.4T1 cells was examined by RT-PCR. The results are presented as the mean ± SEM. Statistical significance was analyzed by unpaired Student’s *t* test. **p < 0.01, *n* = 4. **C** Wnt7a protein levels in LM.4T1 and HM.4T1 cell lysates were evaluated by western blotting. The results are presented as the mean ± SEM. Statistical significance was analyzed by unpaired Student’s *t* test. **p < 0.01, *n* = 4. **D** Wnt7a protein levels in LM.4T1 and HM.4T1 cell-derived exosomes were evaluated by western blotting. **E** Parental 4T1, LM.4T1, and HM.4T1 cells were cultured for 4 days with or without recombinant Wnt7a protein (100 ng/ml). The diameter of each spheroid was measured by the Image J software. Scale bar indicates 100 μm. The results are presented as the mean ± SEM. Statistical significance was analyzed by two-way ANOVA with multiple comparisons. *p < 0.05, **p < 0.01, ****p < 0.0001, *n* = 6. **F** The presence of Wnt7a in exosomes derived from wild type or Wnt7a (−) LM.4T1 was examined by western blotting. **G** Fifty thousand HM.4T1 cells were mixed with the same number of control LM.4T1 cells or Wnt7a (−) LM.4T1 cells and implanted into the 3rd mammary pad of BALB/c nude mice. The size of each tumor was measured, and the tumor volume was calculated. After 28 days of monitoring the tumor. *n* = 7. **H** Four weeks after the injection, tumor tissues were excised and weighted. The results are presented as the mean ± SEM. Statistical significance was analyzed by Student’s *t* test. *n* = 7. **I** The number of metastatic tumor nodules was counted 4 weeks after the injection. The results are presented as the mean ± SEM. Statistical significance was analyzed by unpaired Student’s *t* test. *p < 0.05, *n* = 7

Wagenblast et al. previously isolated subclones from 4T1 cells by marking individual cells with a molecular barcode and then performed RNA-seq analysis on 23 subclones [20]. To discover potential parameters involved in the cooperation between the two 4T1 subclones in this study, we analyzed their RNA-seq data deposited to the Gene Expression Omnibus (GEO) (GSE63180) using the GEO RNA-seq Experiments Interactive Navigator (GREIN) [25]. A correlation matrix displayed the presence of two distinct subgroups in 4T1 cells (Additional file 1: Fig. S4A). Gene enrichment analysis revealed that a set of genes were over-represented in the epithelial phenotype, while another set of genes were in the mesenchymal phenotype (Fig. 5A). *Wisp2*, *Casp4*, *Wnt7a*, *Krt80*, and *Gadd45b* mRNA were the top five genes differentially expressed between the two groups, and *Wnt7a* was one of three genes highly expressed in the epithelial group (Fig. 5A, Additional file 1: Fig. S4B). Wnt7a is a member of the Wnt family and activates both canonical and non-canonical signaling pathways in receptive cells [30, 31], and abnormal Wnt signaling has been found in most cases of BC [15]. Among the Wnt family members, only *Wnt7a* and *Wnt7b* mRNA were expressed at a significant level by the 23 subclones noted above. The expression of *Wnt7b* mRNA was detected in all subclones, whereas the expression of *Wnt7a* mRNA was detected in 43% (10 out of 23) of the subclones (Additional file 1: Fig. S4C). We examined the expression of *Wnt7a* mRNA by LM.4T1 and HM.4T1 cells by RT-PCR and western blotting and found that LM.4T1 cells expressed approximately twofold higher levels of *Wnt7a* mRNA (Fig. 5B) and Wnt7a protein (Fig. 5C) than HM.4T1 cells. Interestingly, Wnt7a protein was detectable in exosomes from LM.4T1 cells, but not from HM.4T1 cells (Fig. 5D). Higher levels of Wnt7a were detected in other 4T1 subclones in the same group (Additional file 1: Fig. S4D). These findings suggested that LM.4T1 cells promote lung metastasis of HM.4T1 cells via exosomal Wnt7a.

### Wnt7a enhances the spheroid formation of HM.4T1 cells and promotes lung metastasis

Wnt7a activates both canonical and non-canonical Wnt signaling pathways via binding to its receptors, resulting in diverse consequences in different diseases and cancers [30–37]. Aberrant Wnt signaling is implicated in TNBC tumorigenesis by its regulation of stem cell self-renewal [38, 39], and cancer stem cells (CSCs) have the capacity to infiltrate and migrate as leader cells, resulting in the initiation and propagation of malignancies [40]. We examined whether Wnt7a could affect the stemness of LM.4T1 and HM.4T1 cells using the sphere formation assay. We cultured parental 4T1, LM.4T1, and HM.4T1 cells in the presence or absence of recombinant Wnt7a protein for up to 4 days and found that Wnt7a could increase the spheroid volume of all three cell types; however, the increase in the size of HM.4T1 spheroids was more significant than that of parental or LM.4T1 cells (Fig. 5E), implying that Wnt7a may enhance the stemness of HM.4T1 cells.

To confirm the involvement of LM.4T1 cell-derived Wnt7a in the increased HM.4T1 cell metastasis, we mutated the *Wnt7a* gene in LM.4T1 cells (Additional file 1: Fig. S5A, Fig. 5F) by the CRISPR/Cas9 technology and implanted them together with HM.4T1 cell into nude (BALB/c-nu/nu) mice. Although we observed no differences in tumor volume and weight (Fig. 5G and H), we did find a decrease in the number of metastatic foci in the lung of mice injected with HM.4T1 cells plus Wnt7a-deficient LM.4T1 cells 4 weeks after the transplantation (Fig. 5I). Since HM.4T1 cells produce a low level of Wnt7a, we also injected a mixture of Wnt7a-deficient LM.4T1 cells and Wnt7a-deficient HM.4T1 cells to completely delete Wnt7a and implanted into nude mice. There was no additional reduction in tumor volume, tumor weight, or the number of metastases (Additional file 1: Fig. S5B, C, D). These results further support our conclusion that LM.4T1 cells enhance lung metastasis of HM.4T1 cells via exosomal Wnt7a.

### Wnt7a activates the Akt/mTORC1 signaling pathway in HM.4T1 cells

**Fig. 6:**
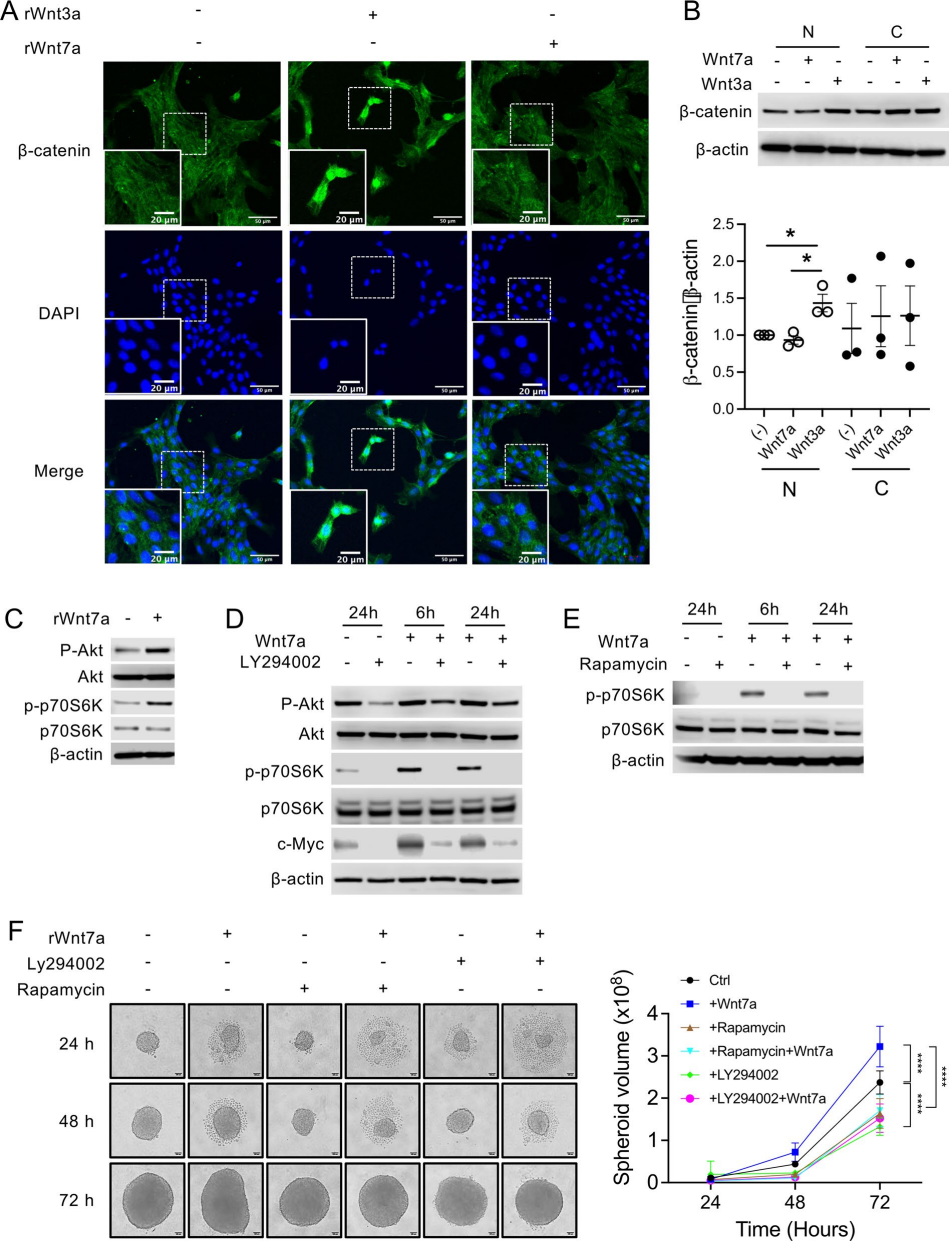
Wnt7a promotes HM.4T1 sphere-forming ability via the PI3K/Akt/mTOR signaling pathway. **A** HM.4T1 cells were grown in 8-well Lab-Tek II chamber slides and stimulated with 100 ng/ml Wnt3a or Wnt7a. Twenty-four hour after the stimulation, nuclear translocation of β-catenin was examined by immunofluorescence. Green: β-catenin. Blue: DAPI. The scale bar indicates 20 μm. **B** HM.4T1 spheroids were stimulated with 100 ng/ml Wnt3a or Wnt7a for 4 days in 3D culture, and the level of β-catenin in the nucleus and cytoplasm was evaluated by western blotting. The results are presented as the mean ± SEM. Statistical significance was analyzed by two-way ANOVA with multiple comparisons. *p < 0.05, *n* = 3. **C** HM.4T1 spheroids were stimulated with 100 ng/ml Wnt7a for 24 h, and then, phosphorylation of Akt and p70S6K was evaluated by western blotting. **D** HM.4T1 spheroids were treated with or without 10 μM LY294002 for 1 h and then incubated for another 24 h. Phosphorylation of Akt and p70S6K or the level of c-Myc was evaluated by western blotting. To examine the effects of LY294002 on Wnt7a-induced phosphorylation of Akt and p70S6K or c-Myc expression, cells in spheroids were lysed after 6 and 24 h. **E** HM.4T1 spheroids were treated with or without 50 ng/ml rapamycin for 1 h and then incubated for another 24 h. Phosphorylation of p70S6K was evaluated by western blotting. To examine the effects of rapamycin on Wnt7a-induced phosphorylation of p70S6K, cells in spheroids were lysed after 6 and 24 h. **F** HM.4T1 spheroids were treated with DMSO or 10 μM LY294002 or 50 ng/ml rapamycin for 1 h and then incubated for up to 72 h in the presence or absence of 50 ng/ml Wnt7a. Photographs were taken at 24, 48, and 72 h. The diameter of each spheroid was measured by the Image J software. Scale bars indicate 100 μm. The results are presented as the mean ± SEM. Statistical significance was analyzed by two-way ANOVA with multiple comparisons. ****p < 0.0001, *n* = 6

We next explored potential signaling pathways by which Wnt7a enhances the migration and subsequent metastasis of HM.4T1 cells. We first used immunofluorescence to detect the nuclear translocation of β-catenin after stimulation with Wnt7a or Wnt3a in 2D culture. Wnt3a was used as a control stimulus that induces the nuclear translocation of β-catenin [41]. As previously reported, Wnt3a induced the nuclear translocation of β-catenin and the level of nucleus β-catenin increased in HM.4T1 cells (Fig. 6A, left and middle columns). By contrast, Wnt7a had no effect (Fig. 6A, right column). We also examined the nuclear translocation of β-catenin after activating the cells with Wnt3a or Wnt7a in 3D culture. We used western blotting since immunofluorescence was not useful to detect nuclear translocation of β-catenin in 3D culture. Again, there was no increase in the level of β-catenin in the nucleus after Wnt7a stimulation (Fig. 6B). These findings indicated that Wnt7a did not activate the canonical Wnt/β-catenin-dependent pathway in HM.4T1 cells.

Wnt7a is also known to mostly favor the PI3K/Akt/mTOR pathway via binding to FZD7 receptor [36]. As shown in Fig. 6C, Wnt7a stimulation increased the level of Akt phosphorylation in HM.4T1 cells. The phosphorylation of p70S6K, a target molecule of mTORC1, also increased, strongly suggesting that Wnt7a acts on HM.4T1 cells via mTORC1. To further clarify the involvement of the PI3K/Akt/mTORC1 pathway, we used an inhibitor of PI3K (LY294002) or mTORC1 (rapamycin). LY294002 significantly blocked the phosphorylation of Akt and p70S6K and the expression of c-Myc that has an ability to regulate the stem-like properties and metastasis of TNBC cells [42] (Fig. 6D). The Akt signaling was reported to influence the expression of c-Myc [43]. Rapamycin also inhibited Wnt7a-induced phosphorylation of p70S6K (Fig. 6E), indicating that Wnt7a activates the PI3K/Akt/mTORC1 pathway in HM.4T1 cells. Finally, we treated HM.4T1 cells with these inhibitors and evaluated the formation of HM.4T1 spheres in 3D culture in the presence or absence of Wnt7a. Both inhibitors significantly reduced the volume of HM.4T1 spheroids at 72 h (Fig. 6F), supporting the conclusion that Wnt7a can enhance the metastasis of HM.4T1 via the PI3K/Akt/mTORC1 signaling pathway.

### LM.4T1 cells contribute to the production of inflammatory TME

**Fig. 7:**
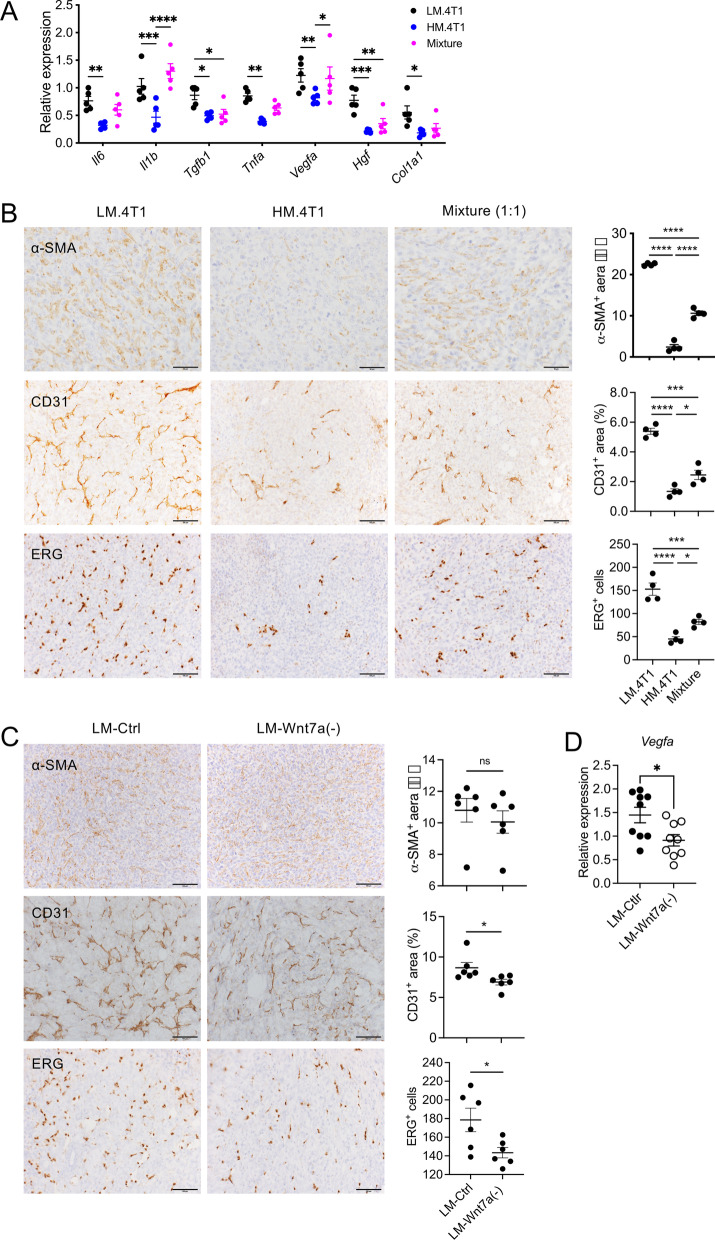
LM.4T1 tumors are highly inflammatory with increased angiogenesis. One hundred and thousand LM.4T1 or HM.4T1 cells were implanted into the 3rd mammary pad of BALB/c mice. Mice were euthanized two weeks later, and tumors were excised for analyses. **A** Expression of *Il6, Il1b, Tgfb1, Tnfa, Vegfa, Hgf, and Col1a1* mRNA was examined by RT-qPCR. The results are presented as the mean ± SEM. Statistical significance was analyzed by two-way ANOVA with multiple comparisons. *p < 0.05, **p < 0.01, ***p < 0.001, ****p < 0.0001, *n* = 5. **B** Cells expressing α-SMA, CD31, or ERG were detected by immunohistochemistry. Percent positive areas for α-SMA- or CD31-positive cells were obtained using the Image J software, and the number of ERG^+^ was counted by eyes. The results are presented as the mean ± SEM. Statistical significance was analyzed by one-way ANOVA with multiple comparisons. *p < 0.05, **p < 0.01, ***p < 0.001, ****p < 0.0001, *n* = 4. **C** One hundred and thousand LM-Ctrl or LM-Wnt7a (−) cells were implanted into the 3rd mammary pad of BALB/c nude mice. Mice were euthanized two weeks later, and tumors were excised for analyses. Cells expressing α-SMA, CD31, or ERG were detected by immunohistochemistry. Percent positive areas for α-SMA- or CD31-positive cells were obtained from two sections, and the number of ERG^+^ was counted by the Image J software. The results are presented as the mean ± SEM. Statistical significance was analyzed by unpaired Student’s t test. *p < 0.05, *n* = 6. **D** Expression of *Vegfa* mRNA was examined by RT-qPCR. The results are presented as the mean ± SEM. Statistical significance was analyzed by unpaired Student’s *t* test. *p < 0.05, *n* = 9

Wnt7a produced by cancer cells was previously demonstrated to recruit and activate fibroblasts and promote tumor aggressiveness of 4T1 cells [44]. Cancer-associated fibroblasts (CAFs) are a major component of tumor microenvironments, and they can promote angiogenesis and provide more nutrients for the growth of tumor cells and to prevent necrosis [45]. Hepatocyte growth factor (HGF) secreted by myofibroblasts was previously shown to enhance the Wnt signaling [46]. To examine a potential role for LM.4T1 cells in the production of microenvironment favorable for cancer cell metastasis, we injected LM.4T1, HM.4T1, or a cell mixture (1:1) into BALB/c mice and examined the expression levels of cytokines at 2 weeks. By RT-qPCR, the expression levels of *Il6*, *Il1b*, *Tgfb1*, *Tnfα*, *Vegfa*, *Hgf*, and *Col1a1* mRNA, which could be expressed by CAFs, were higher in tumors of LM.4T1 or the cell mixture than those of HM.4T1 (Fig. 7A). We next examined the infiltration of CAFs by immunohistochemistry. α-SMA positive cells, potentially CAFs, were more abundant in tumors of LM.4T1 or the cell mixture than those of HM.4T1 (Fig. 7B, top row). A higher level of α-SMA was also detected in tumors of LM.4T1 cells by western blotting (Additional file 1: Fig. S6). The area of CD31^+^ staining and the number of ERG^+^ endothelial cells were higher in tumors of LM.4T1 or the cell mixture than those of HM.4T1, indicating that angiogenesis was more prominent in LM.4T1 tumors (Fig. 7B, middle and bottom rows).

**Fig. 8:**
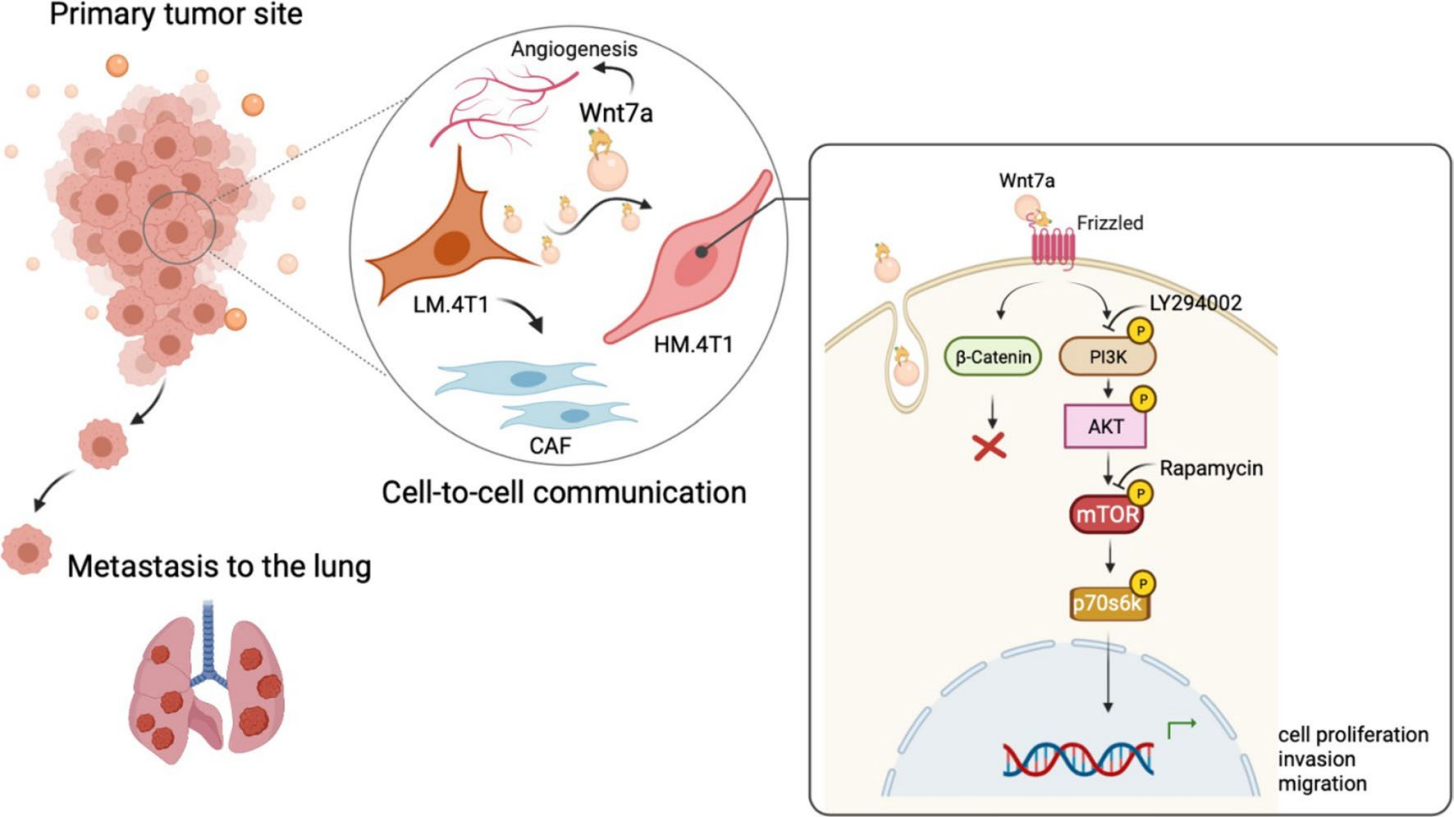
A low metastatic subclone promotes lung metastasis of highly metastatic subclone via exosomal Wnt7a. Tumors comprise multiple subclones with different features. In the TME of 4T1 BC, LM.4T1 cells secrete exosomes containing Wnt7a, leading to the activation of the PI3K/Akt/mTOR signaling pathway and subsequent increases in the stemness and invasion in HM.4T1 cells. LM.4T1-derived Wnt7a also increases angiogenesis, contributing to the increased metastasis by HM.4T1 cells

To examine whether LM.4T1-derived Wnt7a was responsible for the increased CAF infiltration or angiogenesis, we injected LM.4T1 control (LM-Ctrl) or Wnt7a-deficient LM.4T1 (LM-Wnt7a (-)) cells in the mammary pad of nude mice. Although we did not find a difference in the infiltration of α-SMA positive cells, the numbers of CD31^+^ or ERG^+^ cells decreased in tumors of Wnt7a-deficient LM.4T1 cells. The expression of *Vegfa* mRNA was also lower in tumors of Wnt7a-deficient LM.4T1 cells, suggesting that Wnt7a is responsible for the increased angiogenesis in tumors of LM.4T1 cells. The involvement of the PI3K-Akt pathway in the expression of *VEGFA* in human breast cancer cells was previously reported [47]. Thus, Wnt7a secreted by LM.4T1 cells not only acts directly on HM.4T1 cells to enhance their metastatic capacity, but also contributes to the creation of tumor stroma necessary for cancer cell metastasis (Fig. 8).

## Discussion

Intratumor heterogeneity appears in all kinds of cancer and the risk of mortality increased when more than two clones coexisted in the same tumor sample [48]. Many prevalent mutations have been discovered in BC, resulting in genetic and phenotypic diversity. Aneuploid rearrangements occur early in tumor evolution and remain highly stable as tumor masses expand [49, 50]. This implies that each subclone derived from BC may represent a stable biological state, while some subclones may retain the fitness advantage over others by preserving the fitness of the entire tumor or subpopulations. By using the murine TNBC cell line 4T1, we established two distinct subclones with low (LM.4T1) or high (HM.4T1) metastatic potential. In contrast to previous studies showing the ability of highly metastatic cells to assist low metastatic cells in obtaining a metastatic ability [51–53], we discovered that low metastatic cells could assist lung metastasis of highly metastatic cells using at least two mechanisms: by secreting exosomal Wnt7a that increases the capacity of highly metastatic cells via activation of the PI3K/Akt/mTOR signaling pathway and by increasing angiogenesis in the tumor microenvironment for a better escape of highly metastatic cells.

The process of tumor metastasis begins with local invasion of cancer cells in the primary tumor, and epithelial–mesenchymal transition (EMT) is a critical mechanism in cancer cells invasion [54]. Compared to LM.4T1 cells, HM.4T1 cells exhibited a mesenchymal cell morphology with a higher level of Snail expression and metastatic potential, suggesting that HM.4T1 cells are at a more advanced stage of EMT. To further investigate the components that differentiate these two types of cells, we analyzed previously reported RNA sequencing data of 23 individual 4T1 subclones in the GEO database and divided them into two subgroups, epithelial cell-like and mesenchymal cell-like, based on the gene expression correlation matrix. Wnt7a was subsequently discovered to be one of the genes differentially expressed between the groups and expressed significantly higher in epithelial cell-like than the mesenchymal cell-like subgroup. We detected a higher level of Wnt7a in LM.4T1 cells than HM.4T1 cells, leading to a question whether there is a link between Wnt7a expression and EMT. In a previous study, there was a clear correlation between the expression of the EMT suppressor grainyhead transcription factor Grhl2 and the epithelial marker E-cadherin: Grhl2 was significantly down-regulated in disseminated cancer cells that had undergone EMT [55]. In an in vitro human mammary epithelial cell culture model, WNT7A was found to be up-regulated in cell populations expressing epithelial markers and down-regulated in cell populations expressing mesenchymal markers [56]. Similarly, we found that a highly metastatic HM.4T1 cells with mesenchymal cell-like phenotypes expressed a low level of Wnt7a, whereas low metastatic LM.4T1 cells with epithelial cell-like phenotypes expressed a high level of Wnt7a, suggesting a link between Wnt7a expression and EMT. Interestingly, HM.4T1 cells were able to receive a significant amount of Wnt7a from LM.4T1 cells. In a *MMTV-Wnt1* transgenic mouse model, Wnt1 secreted by luminal subtype of breast cancer cells promoted the proliferation of basal-like recipient cells [57]. Given that tumors consisted of diverse subclones, the various kinds of subclones likely interact in a paracrine way using multiple molecules and contribute to the tumor progression.

Extracellular vesicles (EVs) secreted from cancer cells function in cancer cell dissemination by working as modulators of tumor microenvironments. Different kinds of EVs are classified based on their sub-cellular origin [58]. Exosomes, enriched in late endosome components, were defined as a small EV subtype containing the tetraspanins CD63 and CD9/CD81 proteins [59]. So far, tumor cell-derived exosomes have been shown to be involved in nearly all steps of the invasion–metastasis cascade by activating the EMT process in neoplastic epithelial cells, fostering a premetastatic niche in distant organ, and modulating host immunity [60]. Wnt proteins can be transferred by a paracrine mechanism, but it remains unclear how they travel in the extracellular space. In Drosophila, wingless (Wg) is transferred via exosomes by binding to the exosomal protein Evi, implying that Wnt proteins can travel with exosomes [16, 17]. Here, we showed that exosomes from LM.4T1 cells increased the lung metastasis of HM.4T1 cells. When the release of exosomes from LM.4T1 cells was blocked or the *Wnt7a* gene was mutated in LM.4T1 cells, the number of metastases in the lungs of mice injected with a mixture of LM.4T1 and HM.4T1 cells decreased, strongly suggesting that Wnt7a on exosomes was responsible.

Wnt7a can activate both canonical and non-canonical Wnt signaling pathways and play diverse functions in various malignancies. In bladder and oral squamous carcinomas, it promoted cancer cell invasion and migration via a canonical β-catenin-dependent signaling pathway [31, 32], whereas Wnt7a overexpressed in non-small cell lung cancer enhanced tumor radiosensitivity via a non-canonical Wnt/JNK pathway [34]. We observed that when recombinant Wnt7a was added to HM.4T1 cells in 3D cell culture, the volume of tumor spheroids increased significantly. However, we did not detect the accumulation of β-catenin in the nucleus, revealing that Wnt7a did not activate the canonical Wnt signaling pathway. Wnt7a/Fzd7 interaction was shown to maintain muscle stem/progenitor cell expansion via a non-canonical Wnt pathway, called planar cell polarity pathway, driven by VangL2 and induce muscle cell hypertrophy via the PI3K/Akt/mTOR pathway [33, 36]. We detected the activation of the PI3K/Akt/mTOR signaling pathway in Wnt7a-stumulated HM.4T1 cells. The PI3K/Akt/mTOR signaling pathway is a prominent intracellular signaling pathway that plays an important role in tumor cell growth and proliferation [61]. mTOR is a protein kinase that is inactivated by a bacterial toxin, rapamycin. There are two different multiprotein mTOR complexes: mTORC1 and mTORC2. p70S6 kinase is a downstream target of mTORC1, and its target substrate is S6 ribosomal protein. The phosphorylation of p70S6 kinase induces protein synthesis in ribosome [62]. In HM.4T1 cells, a low concentration of rapamycin (50 nM) significantly inhibited sphere formation and constitutive and Wnt7a-induced p70S6K phosphorylation. In BC, p70S6K was associated with angiogenesis and lung metastasis [63, 64], and elevated p70S6K level was found to correlate with a poor prognosis and survival in BC patients [65]. We also discovered rapamycin reduced the phosphorylation of Akt, which can also be activated via mTORC2. A previous study revealed that higher concentrations of rapamycin (0.2–20 μM) could target mTORC2, while lower concentrations (0.5–100 nM) could target mTORC1 [66]. Aside from the effect by different concentrations, chronic rapamycin administration can also impact mTORC2 assembly, which in turn suppresses Akt activation [67]. Since we used a low concentration of rapamycin, we concluded that its effects were on mTORC1. However, additional experiments are needed to clarify the role of mTORC2 in the process.

Angiogenesis is a critical component of cancer metastasis. We detected higher numbers of ERG- or CD31-positive cells with higher *Vegfa* mRNA expression in LM.4T1 tumors than in HM.4T1 tumors, suggesting that LM.4T1 cells have a higher capacity to induce angiogenesis than HM.4T1 cells. This could be another way LM.4T1 cells assist HM.4T1 cell metastasis. We investigated whether Wnt7a was responsible for the higher level of angiogenesis in LM.4T1 tumors and found that tumors of Wnt7a (−) LM.4T1 cells had lower numbers of ERG- or CD31-positive cells with a lower level of *Vegfa* mRNA expression than those of Wnt7a (+) LM.4T1 cells. Although the evidence linking Wnt7a to tumor angiogenesis is rather limited, astrocyte-derived Wnt7a was shown to stimulate angiogenesis [68], suggesting that Wnt7a may play a role in tumor angiogenesis which also contributes to cancer cell metastasis.

To explore the possibility that a similar mechanism is also present in human TNBC, we evaluated the expression of *WNT7A* mRNA in human TNBC cell lines using the preexisting data in the GEO database [69]. Interestingly, a high level of *WNT7A* mRNA expression was often detected in the basal-like subtype of TNBC but not in the mesenchymal-like subtype (Additional file 1: Fig. S7A). We also analyzed the association between the level of *WNT7A* mRNA expression and patients’ prognosis using the existing microarray database and found that higher *WNT7A* mRNA expression was associated with poor overall survival (OS) of all patients with BC (HR 1.24) and patients with basal-like subtype (HR 1.54) (Additional file 1: Fig. S7B) that is often diagnosed as TNBC and comprises about 50 to 75% of the TN subtype [70]. Although it remains unclear whether interclonal communication by WNT7A plays a role in the progression of human TNBC, it is interesting to see the association between *WNT7A* mRNA expression and patients’ survival in human BC cases.

## Conclusions

Therapeutic targets of TNBC are mostly unknown, and chemotherapies are still the primary treatment of TNBC patients [4]. Previous studies mostly targeted the intrinsic features of highly metastatic cancer subclones to control cancer metastasis, but the contribution of low metastatic subclones remains unclear. Here, we analyzed the mechanism of interclonal cooperation using the murine 4T1 TNBC model and found that Wnt7a derived from a low metastatic subclone activates the PI3K/Akt/mTOR signaling pathway and enhances the lung metastasis of a highly metastatic subclone. Wnt7a also increases angiogenesis in tumor stroma. Thus, Wnt7a could potentially increase the risk of lung metastasis in TNBC patients and may be a new target for the treatment of TNBC patients. Although inhibiting this amplifying loop may not completely prevent the lung metastasis of TNBC cells, combination with other treatments may enhance the effectiveness. Better understanding the mechanisms involved in the progression of TNBC using animal BC models is critical to identify a new strategy to treat TNBC patients in the future.

## Supplementary Information

**Additional file 1**. Supplementary information.

## Data Availability

The datasets supporting the conclusions of this article are included within the article (and its additional files).

## Abbreviations

TNBC: Triple-negative breast cancer
TME: Tumor microenvironment
LM: Low metastatic
HM: High metastatic
FBS: Fetal bovine serum
cDNA: Complementary DNA
RIPA: Radioimmunoprecipitation assay
HRP: Horseradish peroxidase
PBS: Phosphate buffered saline
TEM: Transmission electron microscopy
GFP: Green fluorescent protein
RFP: Red fluorescent protein
GEO: Gene Expression Omnibus
GREIN: GEO RNA-seq Experiments Interactive Navigator
SEM: Standard error of mean
