# Supplementary material for: Exosomal Wnt7a from a low metastatic subclone promotes lung metastasis of a highly metastatic subclone in the murine 4t1 breast cancer
Source: Breast Cancer Res. 2022 Sep 12;24:60. doi: 10.1186/s13058-022-01557-5 (PMC9469633; doi:10.1186/s13058-022-01557-5)
Supplement: Supplementary file 1 — Additional file 1. Supplementary information. [file 13058_2022_1557_MOESM1_ESM.pdf]

Supplementary Information

Exosomal Wnt7a from a Low Metastatic Subclone Promotes Lung Metastasis of a Highly Metastatic Subclone in the Murine 4T1 Breast Cancer

Chunning Li[1], Teizo Yoshimura[1*], Miao Tian[1], Yuze Wang[1], Takamasa Kondo[1], Ken-Ichi Yamamoto[2], Masayoshi Fujisawa[1], Toshiaki Ohara[1], Masakiyo Sakaguchi[2] and Akihiro Matsukawa[1*]

[1]Department of Pathology and Experimental Medicine, [2]Department of Cell Biology, Graduate School of Medicine, Dentistry and Pharmaceutical Sciences, Okayama University, 2-5-1 Shikata, Kita-ku, Okayama 700-8558, Japan.

Table S1. Antibodies used in this study

Western blot		
**Primary antibody**		
Protein name	Dilution	Company
CD63	1:1000	Abcam #ab217345
TSG101	1:1000	Abcam #ab125011
GM130	1:500	BD #610822
β-catenin	1:1000	CST #8480
Wnt7a	1:1000	Abcam #ab100792
c-Myc	1:1000	CST #5605
p70S6K	1:1000	CST #9202
Phospho-p70S6K	1:1000	CST #9234
Snail	1:1000	CST #3879
AKT	1:1000	CST #4691
Phospho-AKT	1:1000	CST #9271
Rab27a	1:1000	CST #69295
αSMA	1:200	Dako #M0851
E-cadherin	1:1000	CST #3195
β-actin	1:5000	Sigma #A5441
GAPDH	1:5000	CST #5174
**Secondary antibody**		
Goat anti-rabbit IgG HRP	1:2000	CST #7074
Goat anti-mouse IgG HRP	1:2000	CST #91196
**Immunohistochemistry**		
αSMA	1:200	Dako #M0851
ERG	Undiluted	Nichirei #418111
CD31	1:2000	#ab182981
**Secondary antibody**		
Goat anti-rabbit IgG HRP	Undiluted	Nichirei #414341
Polink-2 Plus HRP anti Mouse Detection Kit	Undiluted	GBI Labs #D37-18
**Immunofluorescence**		
β-catenin	1:3000	CST #19807
**Secondary antibody**		
Alexa Fluor^{TM} 488 goat anti-rabbit IgG (H+L)	1:400	Invitrogen #A-11008

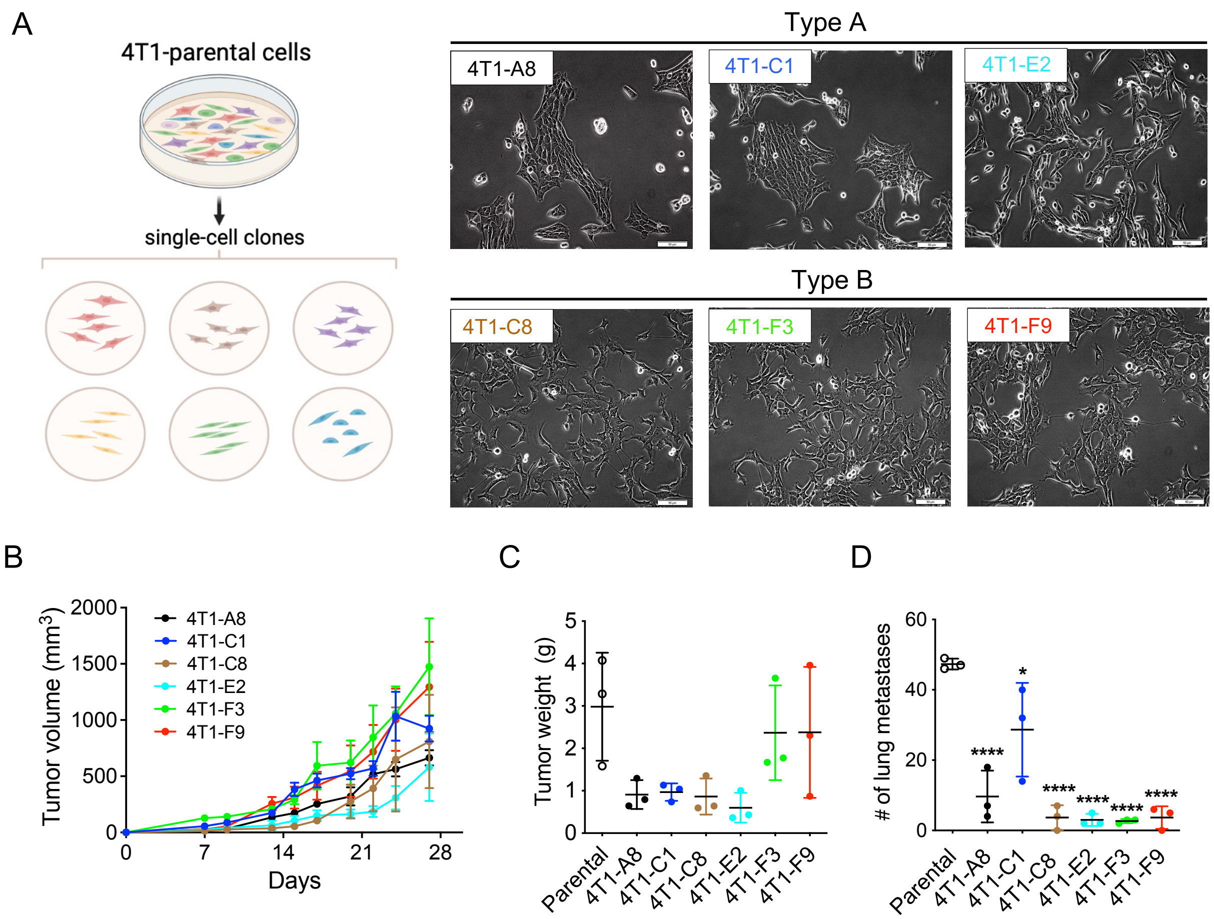

Figure S1. Six single cell clones isolated from parental 4T1 cells show different metastatic potential. **(A)** The cellular morphology of single-cell clones derived from original 4T1 cells by one round of limiting dilution. The scale bar indicates 50 µm. **(B)** One hundred thousand subclone cells were transplanted into the 3rd mammary pad of BALB/c mice. The size of each tumor was measured for 4 weeks and tumor volume was calculated. The results are presented as the mean ± SEM. Statistical significance was analyzed by one-way ANOVA with multiple comparisons. n = 3. **(C)** Mice were euthanized 4 weeks after the injection, and tumor weight was measured. The results are presented as the mean ± SEM. Statistical significance was analyzed by one-way ANOVA with multiple comparisons. n = 3. **(D)** The number of metastases in the lungs was counted. The results are presented as the mean ± SEM. Statistical significance was analyzed by one-way ANOVA with multiple comparisons. *$p < 0.05$, ****$p < 0.0001$, n = 3.

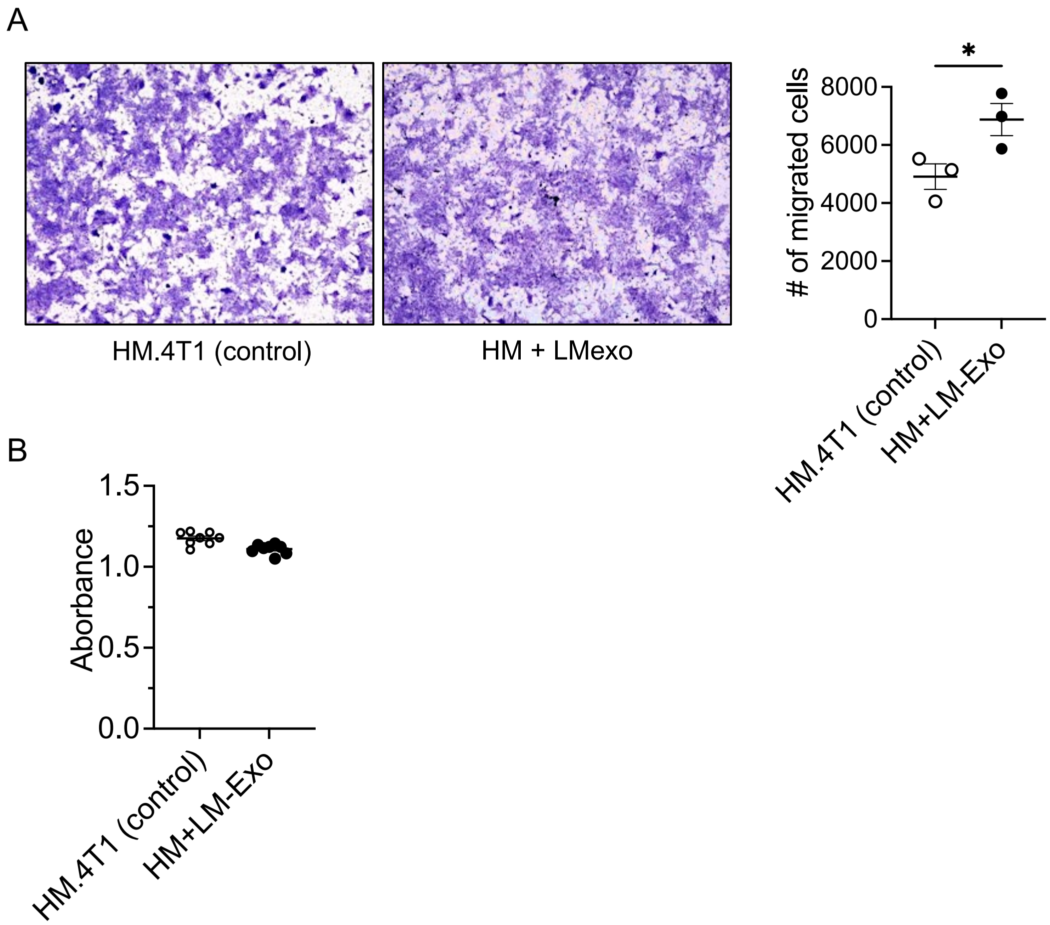

Figure S2. Exosomes from LM.4T1 cells enhance the migration, but not the proliferation, of HM.4T1 cells. **(A)** Transwell migration assay was performed using 24-well inserts with 8-µm pore size (Corning Costar Corp., Corning, NY, USA). HM.4T1 cells suspended in 200 µl of serum-free culture medium were loaded into the inner wells. Exosomes (10µg) isolated from LM.4T1 cells or the same volume of PBS were added to the inner wells. Six hundred µl of culture medium containing 20% FBS was added to the outer wells. After 48 hours, cells on the lower surface of the chamber were fixed with 4% paraformaldehyde for 20 min. After washing 3 times with PBS, the membranes were stained with the Diff Quik Staining kit and photographed under the microscope. The number of migrated cells were counted by using the Image J software. Photos (left panel) and quantitative data (right panel) are presented. n = 3. *$p < 0.05$. **(B)** Twenty thousand HM.4T1 cells were suspended in 1 ml of culture medium that contained a same volume of PBS or LM.4T1 derived exosomes (5 µg), and then 2×10^3 cells were seeded into each well of a 96-well plate. After 4 days of culture, 10 µl of MTT labeling reagent (Roche) was added to each well. After a 4-hour incubation in a humidified environment, 100 µl of the solubilization solution (Roche) was added to each well. Plate was allowed to stand overnight in an incubator in a humidified atmosphere (37℃, 5% CO_2). After complete solubilization of the purple formazan crystals, the spectrophotometrical absorbance of the samples was measured using a microplate reader. The wavelength to measure the absorbance of the formazan product was between 570 and 690 nm. n = 8.

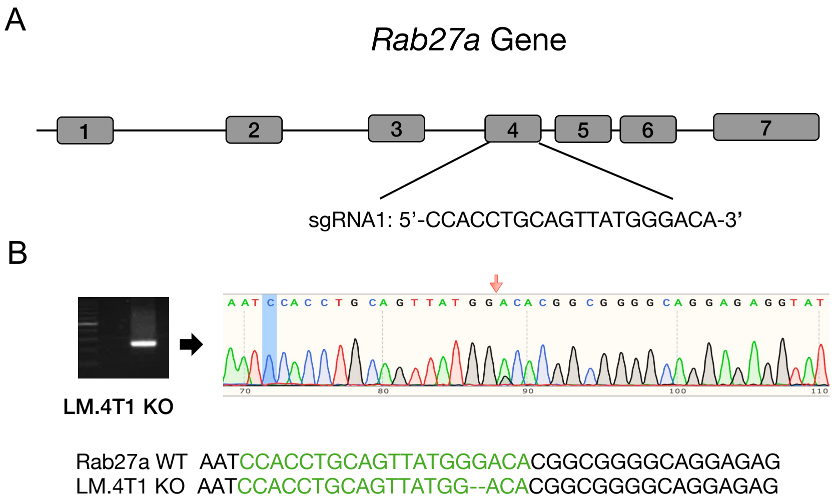

Figure S3. Establishment of stable Rab27a (-) cells by using the CRISPR-Cas9 system. A. The figure illustrates the structure of the mouse *Rab27a* gene, and the guide RNA sequence used to target the fourth exon. **B.** The targeted region was amplified by PCR using genomic DNA isolated from each clone, gel purified and sequenced. The arrow indicates a loss of G in the genomic DNA of LM-Rab27a (-) cells.

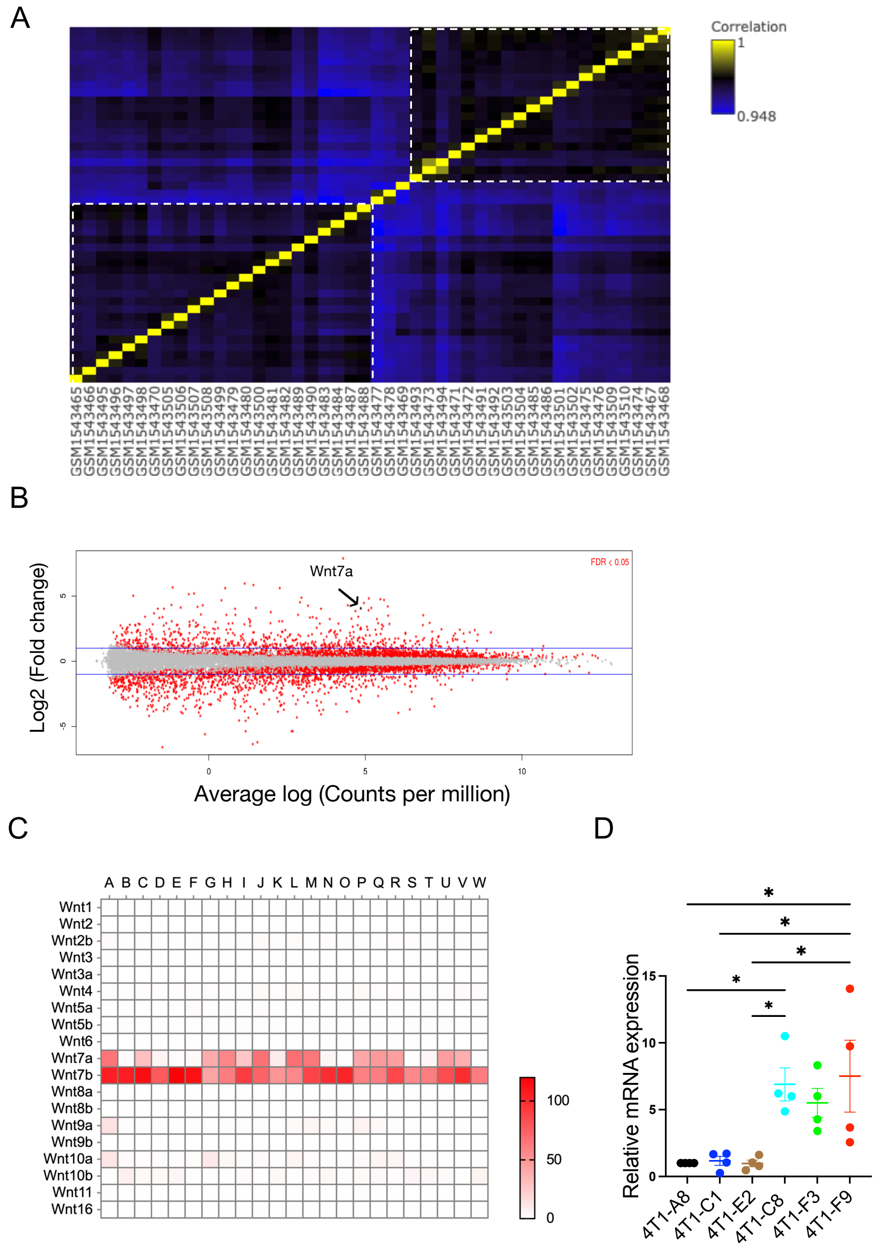

Figure S4. The classification analysis of previously reported 4T1 clonal populations using the GEO RNA-seq Experiments Interactive Navigator (GREIN). (A) A correlation matrix was created using the RNA sequencing data (ID: GSE63180) of 23 subclones isolated from parental 4T1 cells [20]. Two boxes indicate that 23 4T1 subclones could be separated into two subgroups based on the gene expression profile. **(B)** Analysis using the MA plot (log-fold change vs. log-average expression) indicates different gene expression profiles between the two subgroups. False discovery rate (FDR) < 0.05. The arrow points the *Wnt7a* gene. **(C)** The expression levels of genes belonging to the Wnt family by the 23 4T1 subclones are shown by a heatmap. **(D)** The expression of *Wnt7a* mRNA in 6 subclones that we isolated from parental 4T1 cells (presented in Figure S1). The results are presented as the mean ± SE. Statistical significance was examined by ordinary one-way ANOVA with multiple comparisons. n = 4. *$p < 0.05$, **$p < 0.01$.

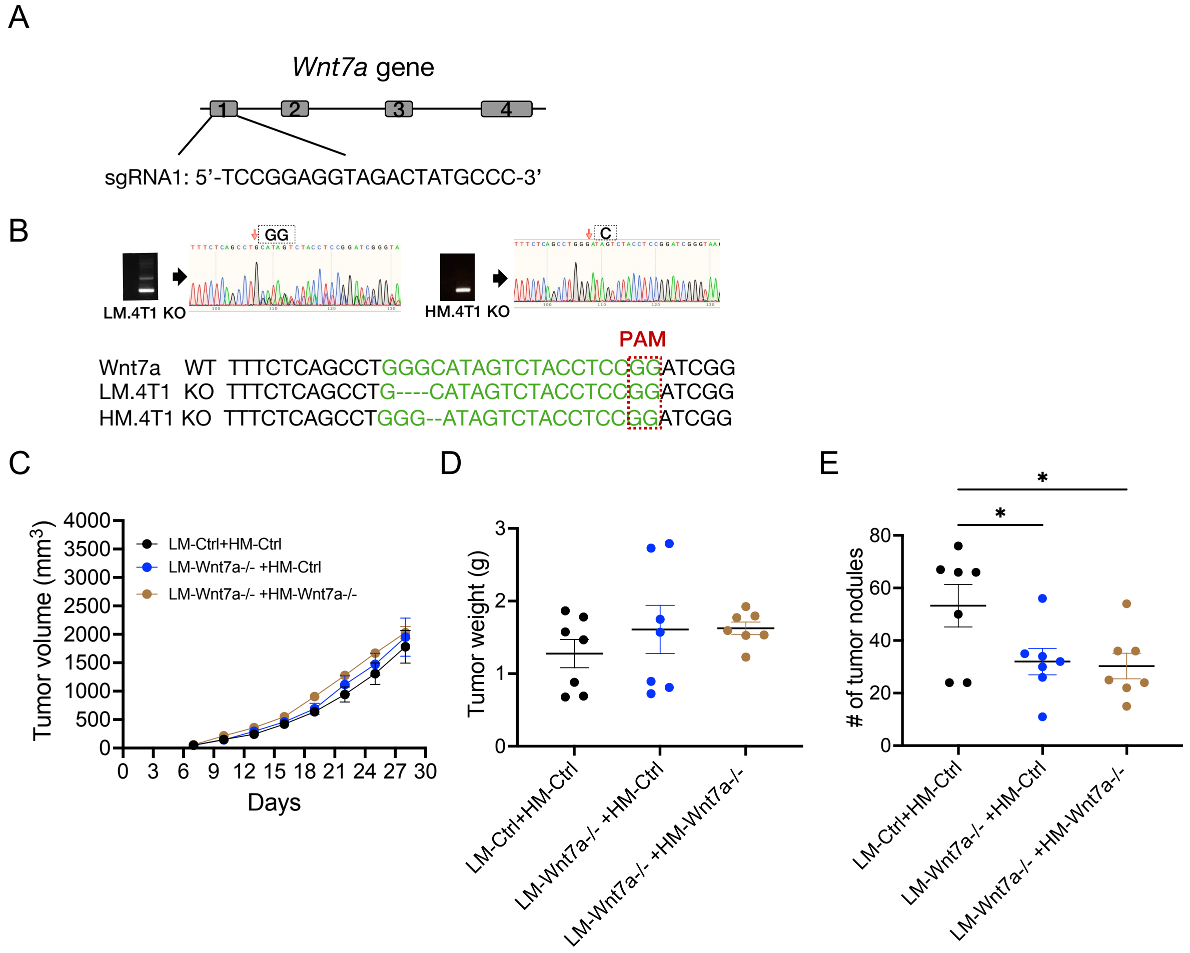

Figure S5. Deletion of Wnt7a in LM.4T1 abrogates the increased HM.4T1 lung metastasis caused by co-injection of LM.4T1 cells. (A) The figure illustrates the structure of the mouse *Wnt7a* gene, and the guide RNA sequence used to target the first exon. **B.** The targeted region was amplified by PCR using genomic DNA isolated from each clone, gel purified and sequenced. In LM-Wnt7a (-) cells, two G are deleted whereas C is deleted in HM-Wnt7a (-) cells. **(C)** 1:1 cell mixture of LM-Ctrl and HM-Ctrl, LM-Wnt7a (-) and HM-Ctrl, and LM-Wnt7a (-) and HM-Wnt7a (-) cells in a total of $1x10^5$ cells were inoculated into the 3rd mammary pad of BALB/c nude mice. Tumor size was monitored for 28 days, and tumor volume was calculated. The results are presented as the mean ± SEM. Statistical significance was examined by ordinary one-way ANOVA with multiple comparisons. n = 7. **(D)** Four weeks after tumor cell inoculation, mice were euthanized, and the weight of each tumor was measured. The results are presented as the mean ± SEM. Statistical significance was examined by ordinary one-way ANOVA with multiple comparisons. n = 7. **(E)** The number of metastases in the lung was counted. The results are presented as the mean ± SEM. Statistical significance was examined by ordinary one-way ANOVA with multiple comparisons. *$p <$ 0.05, n = 7.

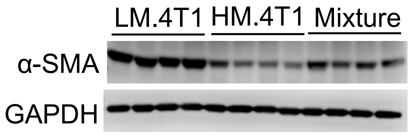

Figure S6. The level of α-SMA is lower in HM.4T1 tumors. Tumor extracts were prepared from tumors of LM.4T1 cells, HM.4T1 cells or cell mixture two weeks after tumor cell inoculation, and the level of α-SMA was examined by western blotting.

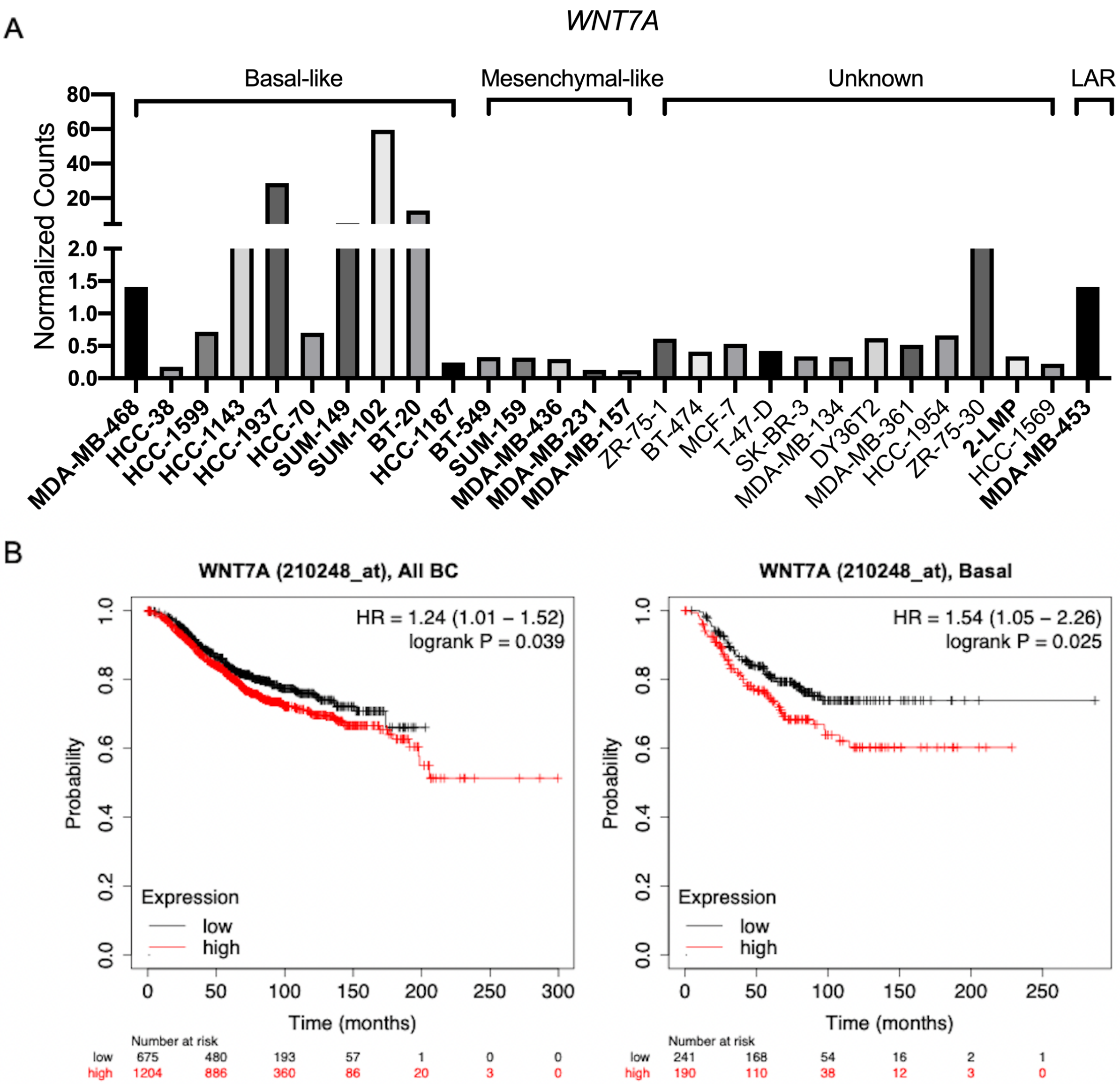

Figure S7. The expression of WNT7 mRNA in human BC cell lines and an association of WNT7A mRNA and the prognosis of patients in six subtypes of TNBC. A. Expression plot. RNA sequencing data of 28 human breast cancer cell lines (10 basal-like, 5 mesenchymal, 12 unknown and 1 LAR) was obtained from the GEO database (ID: GSE58135) and the expression levels of *WNT7A* mRNA were analyzed by using the GREIN [69]. Cell lines indicated by bold letters are TNBC cell lines. **B.** Kaplan-Meier curves generated with the microarray data show that high *WNT7A* mRNA expression in BC, including the Basal-like subtype assigned by PAM50, was associated with poor overall survival (OS) (p = 0.039 and p = 0.025). The plots were generated at https://kmplot.com/analysis/. An auto-cut off was used.
